# Lipid Isobaric Mass Tagging for Enhanced Relative Quantification of Unsaturated *sn*-Positional Isomers

**DOI:** 10.1021/acsmeasuresciau.3c00062

**Published:** 2024-01-11

**Authors:** Tingyuan Yang, Shuli Tang, Jiaxin Feng, Xin Yan

**Affiliations:** † Department of Chemistry, Texas A&M University, 580 Ross Street, College Station, Texas 77843, United States

**Keywords:** lipid *sn*-positional isomer, relative quantification, mass spectrometry, isobaric mass tag, metal adduction

## Abstract

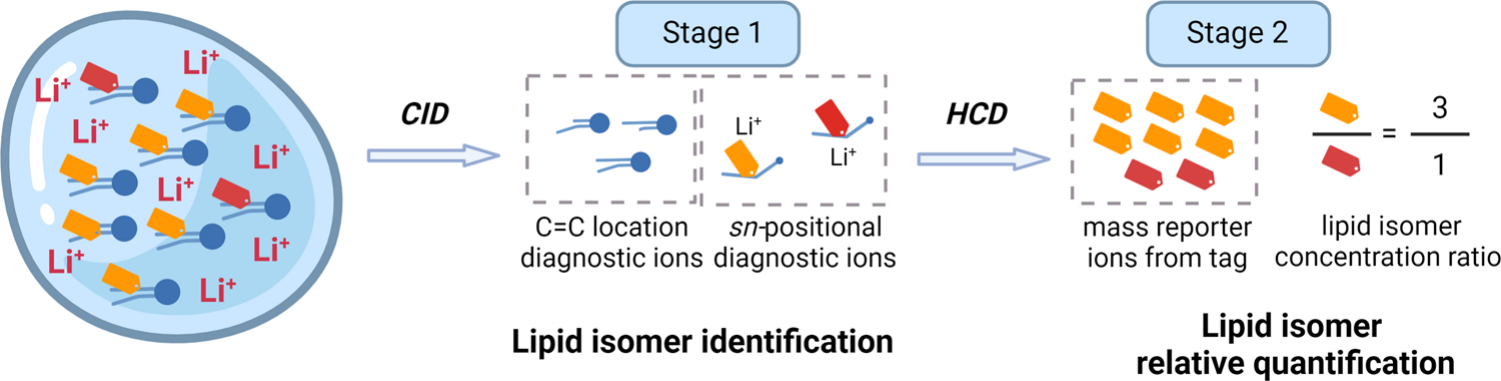

Changes in the levels of lipid *sn*-positional isomers are associated with perturbation of the physiological environment within the biological system. Consequently, knowing the concentrations of these lipids holds significant importance for unraveling their involvement in disease diagnosis and pathological mechanisms. However, existing methods for lipid quantification often fall short in accuracy due to the structural diversity and isomeric forms of lipids. To address this challenge, we have developed an aziridine-based isobaric tag labeling strategy that allows (i) differentiation and (ii) enhanced relative quantification of lipid *sn*-positional isomers from distinct samples in a single run. The methodology enabled by aziridination, isobaric tag labeling, and lithiation has been applied to various phospholipids, enabling the determination of the *sn*-positions of fatty acyl chains and enhanced relative quantification. The analysis of *Escherichia coli* lipid extracts demonstrated the enhanced determination of the concentration ratios of lipid isomers by measuring the intensity ratios of mass reporters released from *sn*-positional diagnostic ions. Moreover, we applied the method to the analysis of human colon cancer plasma. Intriguingly, 17 PC lipid *sn*-positional isomers were identified and quantified simultaneously, and among them, 7 showed significant abundance changes in the colon cancer plasma, which can be used as potential plasma markers for diagnosis of human colon cancer.

Glycerophospholipids (GPLs) are fundamental components of cellular membranes and play diverse roles in cellular functions, encompassing signal transduction, vesicle trafficking, and membrane fluidity regulation.^1−3^ These lipids consist of a glycerol backbone with two fatty acid chains attached to the specific *sn*-1 and *sn*-2 positions on glycerol. *sn*-positional isomers in lipids denote structural variations in the arrangement of fatty acid chains on the glycerol molecule at the *sn*-1 and *sn*-2 positions. Before 2003, there was scarce evidence to suggest the presence of *sn*-positional isomers in eukaryotic cells, even though it is known that both prokaryotic and eukaryotic cells preferentially place unsaturated fatty acids in the *sn*-2 position of GPLs.^4,5^ Ekroos et al. not only confirmed the existence of a measurable population of *sn*-1 unsaturated fatty acyl chains in GPLs but also observed variations in the relative abundance of these *sn*-positional isomers among different mammalian samples.^4^

Remodeling of GPL structures occurs within cells, involving alterations in GPL composition and arrangement.^6^ This process enables cells to adapt to changing environmental conditions and respond to signals, and concurrently gives rise to lipid isomer formation.^7^ The Lands cycle stands out as a pivotal process governing the remodeling of mammalian GPL structures, characterized by the rapid deacylation and reacylation of cellular phospholipids through the action of phospholipase and acyltransferase enzymes.^8^ When two fatty acyl chains attach to different *sn*-1/*sn*-2 positions on the glycerol backbone, lipid *sn*-positional isomers emerge. Perturbations in the physicochemical environment can influence this process, potentially leading to changes in the levels of lipid *sn*-positional isomers in biological systems.^7^ These isomers hold promise as biomarkers for aberrant lipid metabolism in diseases. Recent studies have highlighted shifts in the relative abundance of *sn*-positional isomers in disease contexts, such as during the onset and progression of breast cancer and human lung cancer.^9,10^ These findings underscore the importance of lipid quantification at the isomer level in disease diagnostics and enhance our understanding of pathological mechanisms.

Mass spectrometry (MS) has become indispensable in lipid analysis due to its sensitivity and specificity. While tandem MS, particularly low-energy collision-induced dissociation (CID), has been widely employed for lipid structure analysis, allowing for the assignment of lipid headgroups and fatty acyl chain lengths,^11^ it cannot resolve *sn*-positional isomers and other isomeric forms. Along with other method advancements for isomeric lipid characterization,^12−20^ a few methodologies^21^ have been developed to target *sn*-positional isomer identification. These include charge inversion ion–ion reactions,^22^ anion/cation adduction of lipids,^10,23−26^ radical-induced dissociation of bicarbonate-adducted lipids,^9^ CID fragmentation coupled with ozone-induced fragmentation,^27^ electron-induced dissociation of lipids,^28^ and coupling MS with ion mobility.^29^

Compared with vigorous progress in the characterization of lipid structures, the development of lipid quantification methods has remained relatively stagnant. Current MS-based lipid quantification methods include direct infusion-based shotgun lipidomics,^30^ as well as liquid chromatography (LC)-MS-based approaches, often coupled with selected ion monitoring (SIM), and/or multiple reaction monitoring (MRM).^31^ To achieve enhanced lipid quantification, most of these methods require the use of internal standards (ISs) to compensate for potential variations that may arise during the analysis process, such as fluctuations in analyte ionization conditions due to matrix effects and instrumental variability.^32^ However, acquiring ISs for every lipid in a biological sample is impractical, rendering these methods susceptible to inaccuracies, particularly when it comes to the quantification of lipid isomers.

Isobaric tag labeling, such as iTRAQ (isobaric tag for relative and absolute quantitation) and TMT (tandem mass tag) labeling, becomes a robust quantification method in proteomics, which offers ideal reproducibility, precision, and the ability to multiplex samples without the use of ISs.^33−40^ These tags contain a reactive group for selective protein and peptide modification, a mass balancer, and a mass reporter. Each isobaric reagent comprises a distinctive set of light and heavy isotopes distributed between the balancer and reporter regions. Following the separate labeling of proteins and peptides from various samples, the samples are combined for analysis, effectively eliminating matrix effects. Isobaric tag-labeled proteins/peptides from different samples possess identical masses, while each reporter region produces a unique *m*/*z* reporter ion. The ratios of the reporter ion intensities in tandem MS enable the simultaneous relative quantification of multiple samples within a single run.

In contrast to proteins, lipids exhibit structural diversity, with numerous isomeric forms, and lack common functional groups suitable for tag labeling. Current isobaric tags are only applicable to quantify lipids with amino, carboxyl, and phosphate groups and are not suitable for lipid isomer quantification.^36−40^ Consequently, there is an urgent need for the development of lipid isobaric tags to facilitate the quantification of lipids across various categories at the isomer level. Knowing the prevalence of unsaturated lipids within the overall lipid categories, our previous work reported a lipid isobaric tag labeling strategy that functionalizes lipid C=C bonds via *N*-H aziridination followed by the introduction of isobaric tags (TMT) through *N*-hydroxysuccinimide (NHS) chemistry.^41^ The method allowed us to attain enhanced relative quantification of lipids with the resolution of C=C bond positions. Nonetheless, the method does not offer insights into lipid *sn*-positions, nor does it facilitate quantification of *sn*-positional isomers.

In this study, we have developed an aziridine-based isobaric tag labeling strategy, enabling (i) differentiation and (ii) enhanced relative quantification of lipid *sn*-positional isomers from distinct samples within a single experiment. This involved *N–H* aziridination at the C=C bonds, followed by the incorporation of isobaric iTRAQ tags and the lithiation of tagged lipids from two samples (Figure 1a). Identification of *sn*-positional isomers was accomplished in MS^3^ by detecting diagnostic ions, while MS^4^ facilitated relative quantification through examination of the mass reporter ion ratios released from the two samples (Figure 1b).

**Figure 1:**
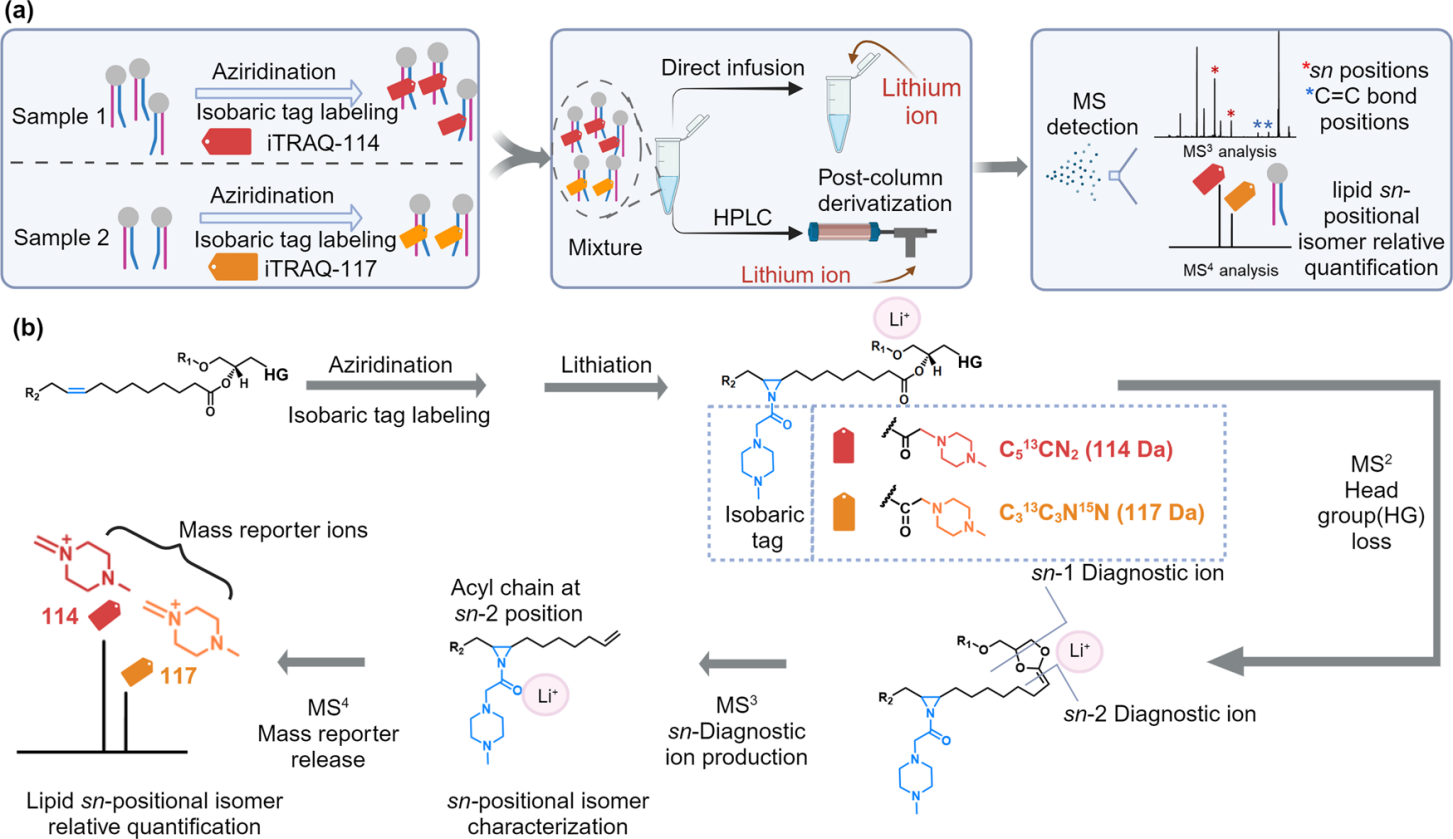
(a) Workflow of lipid *sn*-positional isomer identification and quantification via aziridination, isobaric tag labeling, and lithiation. (b) Structures of lipid modifications through aziridination, isobaric tag labeling, and lithiation as well as the fragment structures of lithium-adducted isobaric tag-labeled lipid after MS^2^, MS^3^, and MS^4^ fragmentation, producing *sn*-positional diagnostic fragments for *sn*-positional isomer identification and mass reporter ions for enhanced relative quantification.

## Experimental Section

### Materials and Reagents

Phosphatidylcholines (PC), phosphatidylglycerols (PG), phosphatidylethanolamines (PE), and *Escherichia coli* lipid extract were purchased from Avanti Polar Lipids (AL). Hydroxylamine-O-sulfonic acid (HOSA) was purchased from Combi-Blocks (CA). Bis [rhodium (α, α,α′,α′-tetramethyl-1,3-benzenedipropionic acid)], formic acid, and lithium acetate were purchased from Fisher Scientific (NH). Pyridine was purchased from Millipore Sigma (MA). Diisopropylethylamine (DIPEA) was purchased from BeanTown Chemical (NH). Ammonium formate was purchased from Sigma-Aldrich (MO). HPLC grade acetonitrile, water, and 2-propanol were purchased from Sigma-Aldrich (MO). Hexafluoro-2- propanol (HFIP) was purchased from CHEM-IMPEX (IL). Human plasma samples were purchased from Innovative Research, Inc. (MI) with a certificate of analysis. iTRAQ-114 and -117 duplex isobaric label reagent set was purchased from Sciex (MA). iTRAQ-113 labeling reagent without isotopic atoms was synthesized in the lab. All solvents and chemicals were used without further purification.

### Lipid Extraction from Human Plasma

PC lipids from human plasma were extracted as follows: 1 mL of methanol was added to 50 μL plasma. The mixture was vortexed for 1 min and then centrifuged at 15,000 rpm for 10 min. The solution phase was collected and dried under a nitrogen stream to obtain the lipid extract for analysis.^42^

### General Procedure for Aziridination

Lipid standards were dissolved in HFIP and mixed with HOSA (1.5 equiv), pyridine (5 equiv), and Rh_2_esp_2_ (20 mol %), and the equivalent refers to the C=C bond amount.^43^ For lipid mixtures, *E. coli* lipid extract was separately dissolved in HFIP with the concentrations of 500 and 1000 μM and mixed with 1.5 mM HOSA, 5 mM pyridine, and 50 μM Rh_2_esp_2_. Human lipid extract was dissolved in 200 μL of HFIP and mixed with 5 mM HOSA, 15 mM pyridine, and 50 μM Rh_2_esp_2_. After stirring the reaction mixture at room temperature overnight, it was prepared for the subsequent isobaric labeling reaction.

### General Procedure for Isobaric Labeling Reaction

The aziridination reaction mixture was directly used for isobaric tag labeling without purification in quantification experiments. The aziridination solution was transferred to a vial for tag labeling. A rotary evaporator was used to remove the solvent. Note that HFIP can react with isobaric tags, and the vacuum was applied to remove HFIP residue. To the vials, acetonitrile was added, followed by the addition of iTRAQ tags (50 equiv for lipid standard and 10 mM for a biological sample). In one vial, iTRAQ-114 was added, while iTRAQ-117 was added to the other (114 and 117 indicate the *m*/*z* of the mass reporters). Both reaction mixtures were stirred at 50 °C for 6 h and then mixed in equal volumes for tandem MS analysis.

### Lithiation Prior to Direct Infusion MS and Postcolumn Lithiation in HPLC-MS

In the context of direct infusion MS analysis, the lithiation process unfolds as follows: after the combination of two samples of tagged lipids, lithium acetate is introduced directly into the sample. The concentration of lithium acetate within the sample is maintained at 100 μM.

For HPLC-MS analysis, a postcolumn lithiation protocol is used. Here, 4 mM lithium acetate is introduced into the flow path postcolumn, achieved through a tee-union setup at a consistent flow rate of 20 μL/min. This infusion of lithium acetate serves to facilitate the formation of the lithium adducts.

### Mass Spectrometry

Mass spectrometric analysis was performed on an Orbitrap Velos Pro (Thermo Fisher Scientific) mass spectrometer. For lipid standards, samples were ionized using a homemade wire-in-a-capillary nanoelectrospray ionization source (nano-ESI) with a spray voltage of around 1.5 kV. The following MS parameters were used for data acquisition: S-lens RF level was set to 67.9%, and the capillary temperature was set at 280 °C. Full MS scans were acquired at *m*/*z* 200–1300 at a resolving power of 30,000 (*m*/*z* 400). A maximum injection time of 500 ms and 1 microscan were used for full MS scans. For lipid identification, tandem MS acquisitions were performed upon CID. CID/CID/HCD fragmentation was needed for the quantification. The HCD energy used for fragmentation was optimized to 60 manufactural units. For lipid isomer quantification from the *E. coli* lipid extract and human plasma, HPLC-ESI-MS was used for analysis. Sheath gas, auxiliary gas, and sweep gas flow rate were set as 35, 10, and 1 arb, respectively. The ionization voltage was 4.5 kV, and the capillary temperature was set to 275 °C. S-lens RF Level was 60%. The raw mass spectrometry data can be found in MetaboLights (https://www.ebi.ac.uk/metabolights/) under the identification number MTBLS8993.

### HPLC Analysis

A vanquish UHPLC system (Thermo Fisher Scientific) coupled with an Orbitrap Velos Pro mass spectrometer (Thermo Fisher Scientific) was applied for LC-MS analysis of the *E. coli* lipid extract and human plasma. An aliquot of 5 μL of the sample was injected into the Accucore C30 column (Thermo Fisher Scientific, C30, 2.1 mm × 150 mm, 2.6 μm). The mobile phase comprised acetonitrile/water (60/40, v/v) (solvent A) and isopropanol/acetonitrile/water (90/10/1, v/v/v) (solvent B), both prepared based on volume ratios. Both contained 10 mM ammonium formate and 0.1% formic acid. The column separation was carried out at 30 °C at a flow rate of 0.2 mL/min. The elute gradient was as follows: 30% B at 0–3 min, 30–43% B at 3–8 min, 43–50% B at 8–9 min, 50%–90% B at 9–18 min, 90–99% B at 18–26 min, 99% B at 26–30 min, and 30% B at 30–35 min.

## Results and Discussion

### Development of an Isobaric Tag Labeling Strategy for Enhanced Relative Quantification of *sn*-Positional Isomers

To achieve enhanced quantification of *sn*-positional isomers, the design of a lipid labeling strategy hinges on two pivotal prerequisites: (i) the acquisition of *sn*-positional diagnostic ions upon CID, and (ii) the inclusion of isobaric tags within these *sn*-positional diagnostic ions, facilitating the subsequent release of mass reporter ions for the quantification of *sn*-positional isomers. In this work, we harness the synergistic potential of aziridine-based isobaric tag labeling for enhanced quantification and metal ion adduction for *sn*-positional isomer identification. This strategic fusion enables us to achieve an enhanced relative quantification of lipid *sn*-positional isomers.

Figure 1a outlines the workflow of the experiment. Lipids from two samples underwent *N*-H aziridination at the C=C bonds followed by the introduction of isobaric iTRAQ tags for quantification purposes. Notably, two isobaric tags (highlighted in red and orange in Figure 1b) were used, which shared the same chemical structure but differed in isotope atoms in mass reporter ions (C_5_^13^CN_2_ at *m*/*z* 114 and C_3_^13^CN^15^N at *m*/*z* 117, respectively). After being labeled, the lipids from the two samples were combined, and lithium ions were added to the mixture (Figure 1a). The mixture was then subjected to either direct infusion MS or HPLC-MS with postcolumn lithiation. Upon CID, the lithiated isobaric-tagged lipid fragmented into a dioxolane due to headgroup loss (Figure 1b). MS^3^ of the dioxolane-induced cross-ring cleavage, providing position-specific fragments for the identification of *sn*-positional isomers. Importantly, these diagnostic fragments of acyl chains carried isobaric tags, which could release mass reporter ions at *m*/*z* 114 and 117 from the two samples in MS^4^ upon higher energy C-trap dissociation (HCD), and their intensity ratios were used to achieve enhanced relative quantification of *sn*-positional isomers. It is worth noting that the diagnostic fragments used to locate C=C bonds could also be generated in CID.

**Figure 2:**
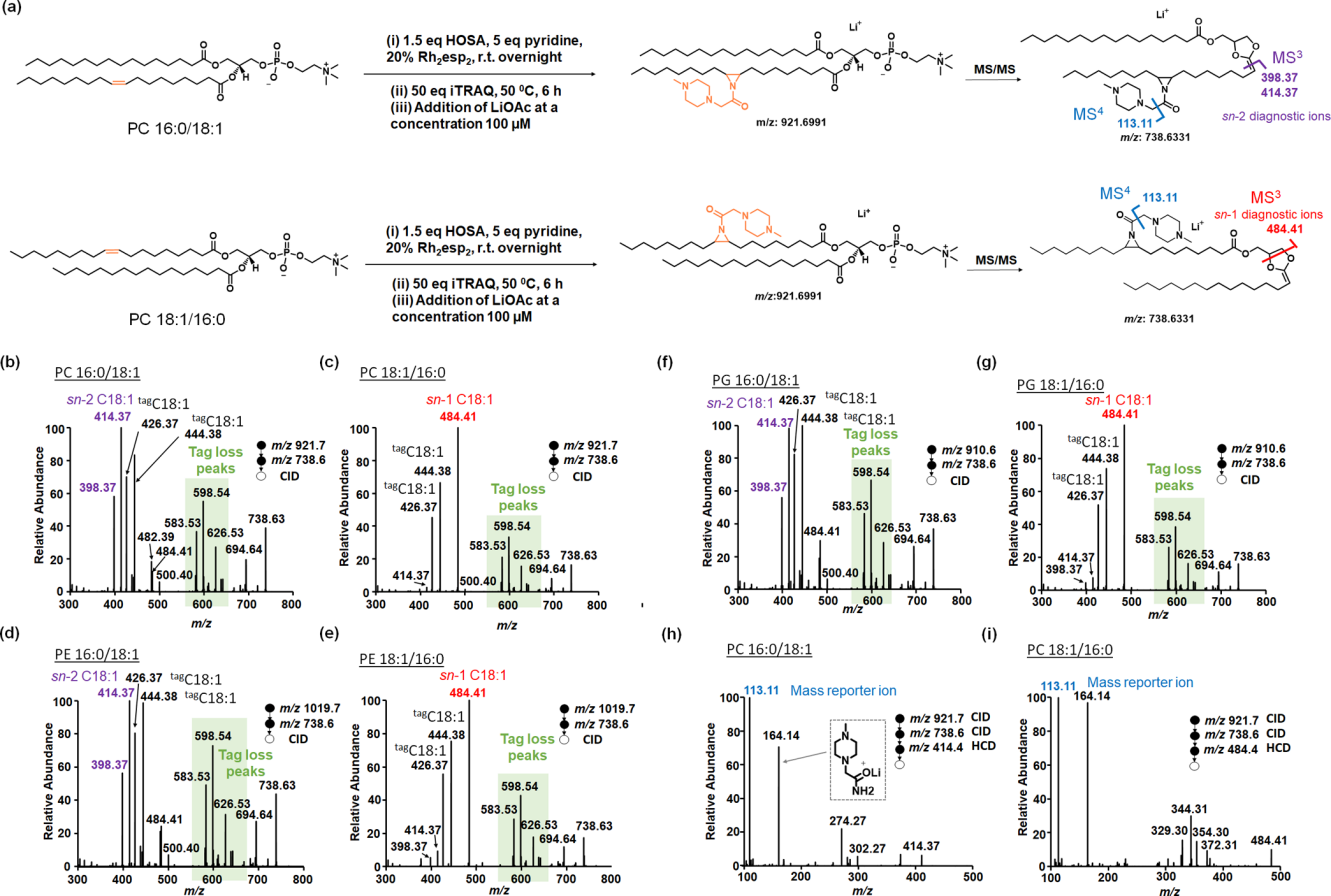
(a) Conditions of lipid derivatization via aziridination and tag labeling followed by CID fragmentation of lithiated tagged lipid PC 16:0/18:1 and PC 18:1/16:0, producing *sn*-1/*sn*-2 positional diagnostic ions and mass reporter ions for *sn*-positional isomer identification and relative quantification. MS^3^ spectra of lithiated tagged lipids: (b) PC 16:0/18:1, (c) PC 18:1/16:0, (d) PE 16:0/18:1, (e) PE 18:1/16:0, (f) PG 16:0/18:1, and (g) PG 18:1/16:0. MS^4^ spectra of lithiated tagged lipids (h) PC 16:0/18:1, (i) PC 18:1/16:0. The mass reporter ions are labeled in blue. *sn*-1 and *sn*-2 positional diagnostic ions are labeled in red and purple, respectively. Tag loss peaks are shaded in green.

We initiated our study by demonstrating that lipids could produce diagnostic fragment ions for *sn*-positional isomer identification upon CID, and release expected mass reporter ions for the purpose of quantification upon HCD after functionalization through *N*-H aziridination, iTRAQ-113 labeling, and lithiation. A pair of *sn*-positional isomer standards, PC 16:0/18:1 and PC 18:1/16:0 was utilized and derivatized to form iTRAQ tag-labeled lipid aziridines. They were then mixed with lithium acetate in a 10 mol equivalent ratio prior to tandem MS analysis (Figure 2a). Upon CID fragmentation of lithiated tagged PC 16:0/18:1 and PC 18:1/16:0 at *m*/*z* 921.7, the dominant fragment corresponding to the headgroup loss ion was detected at *m*/*z* 738.6 in both lipids. Following the MS^3^ fragmentation of this ion, different fragmentation spectra were shown for these two isomers (Figure 2a). *sn*-Positional diagnostic ions were detected at *m*/*z* 398.4 and 414.4 for PC 16:0/18:1 and 484.4 for PC 18:1/16:0 (Figure 2b,c). The structures of the diagnostic ions are shown in Figure S1. Similar to PC lipids, PE (Figure 2d,e) and PG (Figure 2f,g) lipids were also studied and their corresponding *sn*-positional diagnostic ions were detected. Fragmentation of the *sn*-positional diagnostic ions upon HCD released the mass reporter ions, which were detected at *m*/*z* 113 in MS^4^ (Figure 2h,i). The reporter ions were used for lipid isomer quantification in the next experiment.

Importantly, the mass reporter ions were not observed when metal ions other than lithium, such as sodium ions, were employed for adduction. To illustrate this point, the same pair of lipids underwent a similar functionalization process, including aziridination and iTRAQ tag labeling but sodiation. This resulted in the formation of sodiated and tag-labeled lipids at *m*/*z* 937.7. Although tandem MS analysis of these lipids revealed the presence of headgroup loss ions at *m*/*z* 754.6 and *sn*-positional diagnostic ions at *m*/*z* 414.4 and 430.3 for PC 16:0/18:1, and 500.4 for PC 18:1/16:0 (Figure S2), it is worth noting that mass reporter ions were conspicuously absent during the subsequent fragmentation of these diagnostic ions. This absence was primarily attributed to the relatively low ion intensities of the *sn*-diagnostic ions. In contrast, the introduction of lithium-adduction resulted in significantly higher intensities of diagnostic ions and obvious presence of mass reporter ions compared with sodium adduction. This crucial distinction met the prerequisites for both *sn*-positional isomer identification and quantification.

### Reaction Condition Optimization of Aziridination and iTRAQ Labeling

To improve the functionalization efficiencies for lipids with multiple C=C bonds, we optimized reaction conditions for *N*-H aziridination in activating C=C bonds and the iTRAQ labeling process. Our previous study used ethyl 3,3,3-trifluoropyruvate to catalyze *N*-H aziridination of C=C bonds in unsaturated lipids in HFIP solvent.^41^ This reaction exhibits low yields in the case of polyunsaturated lipids, such as an aziridination conversion of 50% for triglyceride 18:1/18:1/18:1.

In this study, we replaced ethyl 3,3,3-trifluoropyruvate with a Rh_2_esp_2_ catalyst, leading to significantly improved aziridination conversion for lipids containing multiple C=C bonds, exceeding 90%. Given its capacity to simultaneously activate multiple C=C bonds in unsaturated lipids, different degrees of aziridination products are expected to be observed (e.g., mono- and diaziridination products). A polyunsaturated lipid standard PC 16:0/20:4 was used to optimize the reaction conditions. We investigated the concentrations of base, nitrogen source, catalyst, and reaction duration (Figure S3). The optimal condition was determined to be as follows: 5 eq pyridine, 1.5 eq HOSA, and 20% eq catalyst relative to the concentration of C=C bonds. The *N*-H aziridination of lipids is complete overnight at room temperature. Notably, the conversion toward a diaziridination product is favored with the conversion rate of 90%.

To achieve the completion of iTRAQ tag labeling of aziridinated lipids, we investigated the amount of iTRAQ, reaction temperature, and reaction time. We found that lipid aziridines could be completely consumed after reacting with 50 equiv of iTRAQ at 50 °C for 6 h (Figure S4).

### Enhanced Relative Quantification of Lipids and Lipid *sn*-Positional Isomers

**Figure 3:**
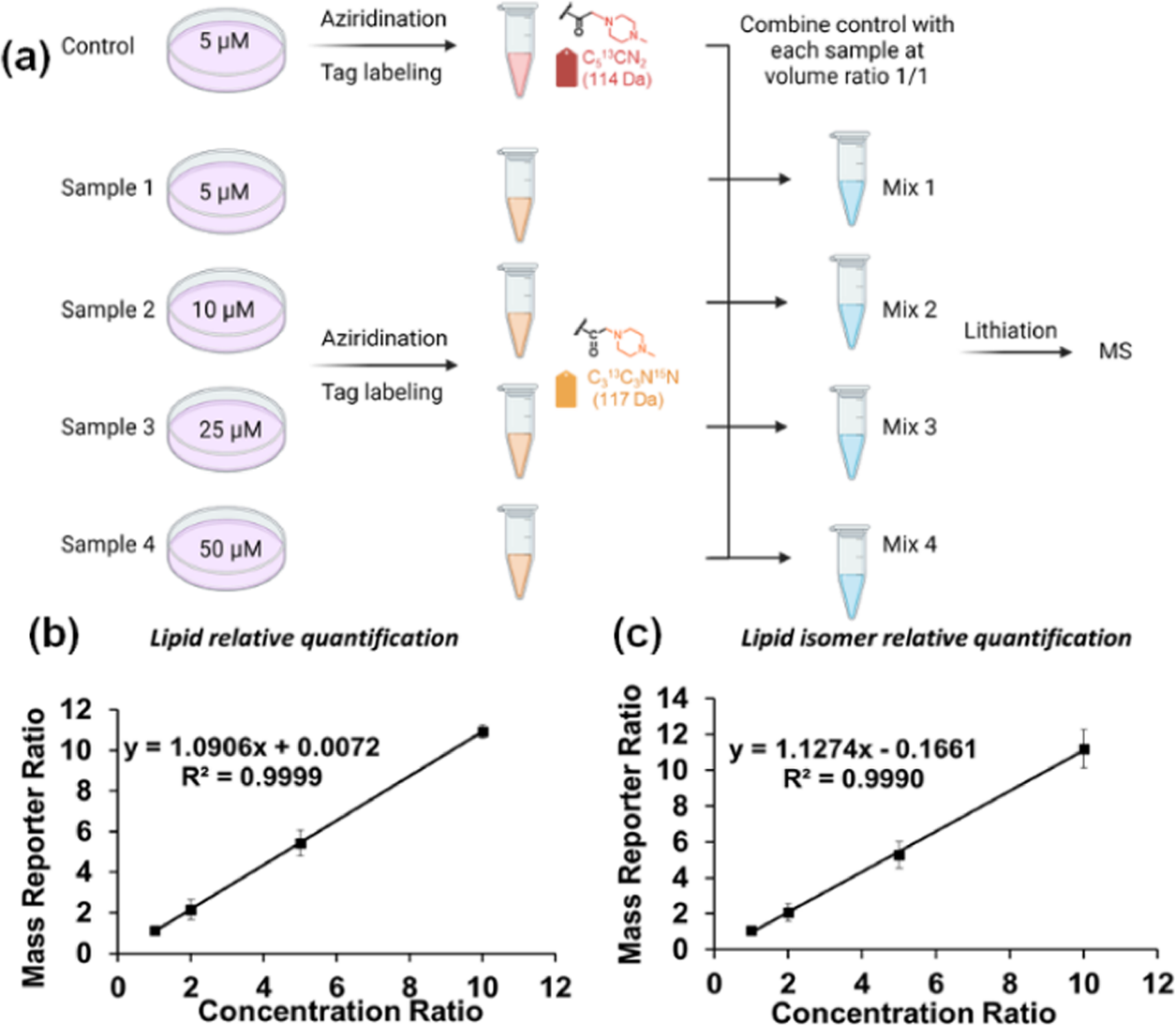
(a) Experimental workflow to create calibration curves for relative quantification of lipid PC 18:1/16:0 at the lipid level (without specifying *sn*-positional isomers) and at the lipid sn-positional isomer level. Control lipid solution was prepared at 5 μM and labeled with iTRAQ-114, while sample solutions were prepared at the concentrations of 5, 10, 25, and 50 μM, and were labeled with iTRAQ-117 separately. Each lipid solution was mixed with the control solution at a volume ratio of 1:1 and subjected to nano-ESI-MS/MS after lithiation. (b) Relative quantification curve at the lipid and (c) relative quantification curve at the *sn*-positional isomer level. Linear correlation was achieved between the intensity ratios of the reporter ions (I114: I117) and the lipid concentration ratios. The reporter ions were obtained upon HCD of tagged lipids in MS^2^ for lipid level quantification and of *sn*-positional diagnostic ions in MS^4^ for *sn*-positional isomer level quantification. Triplicates were performed to generate the error bars.

Taking advantage of the isobaric tags that are introduced to *sn*-positional diagnostic ions, our isobaric labeling strategy is expected to achieve enhanced relative quantification of not only lipids but also *sn*-positional isomers simultaneously. To demonstrate relative quantification capability at both levels, a series of PC 18:1/16:0 lipid solutions were prepared at concentrations of 5, 10, 25, and 50 μM. They were labeled by iTRAQ-117 separately (Figure 3). A control PC 18:1/16:0 solution was prepared at 5 μM and labeled with iTRAQ-114. Each lipid solution was mixed with the control solution at a volume ratio of 1:1 and subjected to nano-ESI-MS/MS after lithiation. Mass reporter ions at *m*/*z* 114 and 117 were released in tandem MS, indicative of the lipids originating from either the lipid solution or the control solution. Notably, the mass reporter ions were released upon HCD in both MS^2^ and MS^4^. The mass reporter ions released in MS^2^ were directly from tagged lipids at *m*/*z* 925.7 so suitable for lipid quantification. In contrast, the mass reporter ions released in MS^4^ were from the *sn*-positional diagnostic ions at *m*/*z* 488.4, thus could be used to quantify *sn*-positional isomers. The correlation between measured ratios of mass reporters and expected concentration ratios of the lipid is displayed in Figure 3. Both show excellent linear relationship with a slope of 1.09 and *R*^2^ of 0.9990 for lipid relative quantification, and a slope of 1.13 and *R*^2^ of 0.9999 for lipid isomer relative quantification. These results indicate that the aziridine-based isobaric tag labeling is feasible to provide enhanced relative quantification of lipids and lipid isomers from two samples in a single MS/MS experiment without using ISs or establishing calibration curves. The limit of detection was determined to be 1 μM for PC 18:1/16:0 and 5 μM for PE 16:0/18:1 and PG 16:0/18:1 after multiple stages of fragmentation to release reporter ions for isomer analysis (Figure S5). This detection threshold may render it challenging to quantify lipid isomers with low abundances. To address this limitation, our future work will involve sample preconcentration before lipid derivatization.

### Generality of Isobaric Tag Labeling for Enhanced Relative Quantification of *sn*-Positional Isomers Across Various Lipid Classes

To assess the versatility of the method in quantifying lipid classes, we extended our investigation to include PE 18:1/16:0 and PG 18:1/16:0, examining quantification at both the lipid level and the lipid isomer level. We applied identical reaction conditions to PE and PG lipid quantification experiments. Lipid samples were prepared at concentration ratios of 1:1, 1:2, 1:5, and 1:10. Subsequent to aziridination and isobaric tag labeling, reaction solutions were combined and diluted to a final concentration of 10 μM, followed by the addition of lithium acetate at a concentration of 100 μM and MS/MS analysis.

The lithiated tagged lipid ions were observed at *m*/*z* 914.6 and 1027.8 for PG and PE, respectively. Upon CID, the predominant features in the spectra were the headgroup loss peaks at *m*/*z* 742.6 for both lipids. Further CID revealed the appearance of *sn*-positional diagnostic ions at *m*/*z* = 488.4 (Figure S6). Upon HCD of these *sn*-positional diagnostic ions, mass reporter ions at *m*/*z* 114 and 117 were released in MS^4^, facilitating isomer quantification (Figure S7). Mass reporter ions that were directedly released from lithiated tagged lipids at *m*/*z* 114 and 117 in MS^2^ were used for quantification at the lipid level. Linear calibration curves were successfully established for both lipid level quantification (Figure S8) and lipid isomer level quantification (Figure S8), with the slope and *R*^2^ values both close to 1 for PE and PG lipids. These results underscore the method’s capability in quantifying both lipids and lipid isomers across lipid classes.

It is noteworthy that PE possesses a primary amino group in its headgroup, which can also react with iTRAQ. Consequently, after aziridination and tag labeling of PE lipids, the dominant product peak in the full spectrum corresponded to the ditagged product. This indicates that both amino groups within the aziridine ring and the headgroup were successfully labeled, rendering the product suitable for lipid quantification (Figure S9).

Furthermore, we studied the method’s quantification capability for lipids containing multiple C=C bonds. PC 16:0/18:2 containing two C=C bonds was used as a model lipid standard. The monoaziridination product was efficiently formed as a dominant peak in the mass spectrum (Figure S10). Subsequently, after isobaric tag labeling, the monoaziridine-monotagged lipid was detected and selected for fragmentation. The observed linear relationship between concentration ratios and mass reporter intensity ratios shows the method’s suitability for quantifying *sn*-positional isomers of polyunsaturated lipids (Figure S8).

### Enhanced Relative Quantification of Lipid Isomers Extracted in*E. coli*

**Figure 4:**
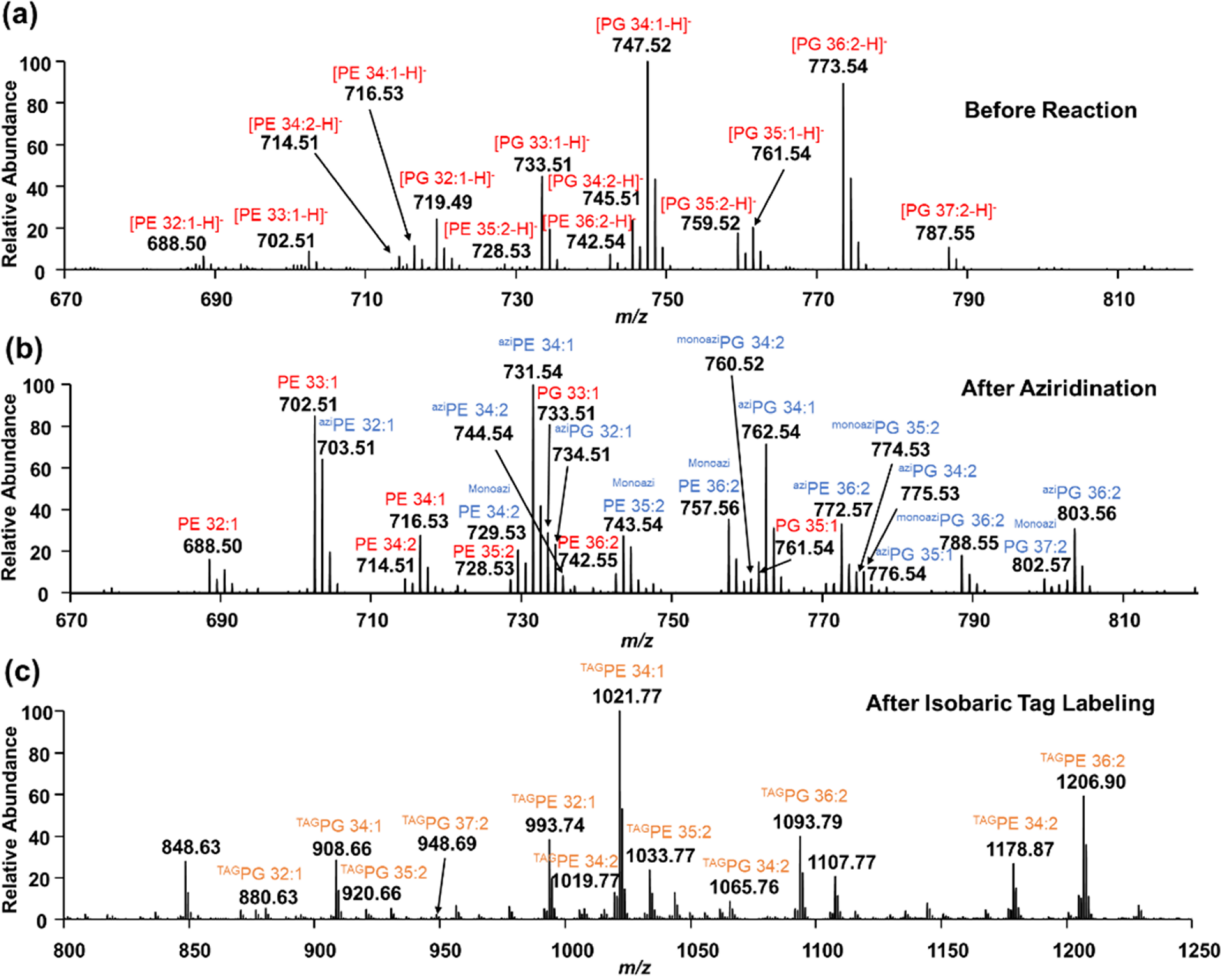
Full mass spectra of the *E. coli* lipid extract (a) before any reactions in negative ion mode, (b) after aziridination in negative ion mode, and (c) after isobaric tag labeling in positive ion mode. Peaks of intact lipids, lipid aziridines, and isobaric tag-labeled lipids are colored red, blue, and orange, respectively. Superscripted monoazi indicates only one C=C bond was aziridinated, while superscripted azi indicates all C=C bonds in the lipids were aziridinated.

To demonstrate the efficacy of aziridine-based isobaric labeling and the accuracy of lipid quantification in biological samples, we performed structural characterization and relative quantification of lipids in *E. coli* samples with predetermined concentration ratios. We prepared the two samples of *E. coli* lipid extract solutions at concentrations of 500 and 1000 μM. Prior to the chemical reactions, 14 glycerophospholipids were detected in negative ion mode (Figure 4a). After aziridination, we observed the corresponding aziridine products in the full mass spectrum in negative ion mode (Figure 4b). It is noteworthy that lipid aziridine products at varying aziridination degrees were present. For instance, both monoaziridination and diaziridination products were detected for PE 34:2, PE 36:2, PG 34:2, and PG 36:2 (Figure 4b). Additionally, our study revealed that monounsaturated lipids PE 33:1 and PG 33:1 did not yield aziridination products. This divergence can be attributed to the presence of cyclopropane moieties in the fatty acyl chains of these lipids, rather than the typical C=C bonds, which was consistent with previous studies.^44^*E. coli* can convert C=C bonds into cyclopropyl groups by adding a methylene group across the C=C bonds of unsaturated fatty acids in phospholipids when exposed to adverse environmental conditions. Lipid C=C bond positions were identified through the CID fragmentation of the lipid aziridines (Figure S11).

**Figure 5:**
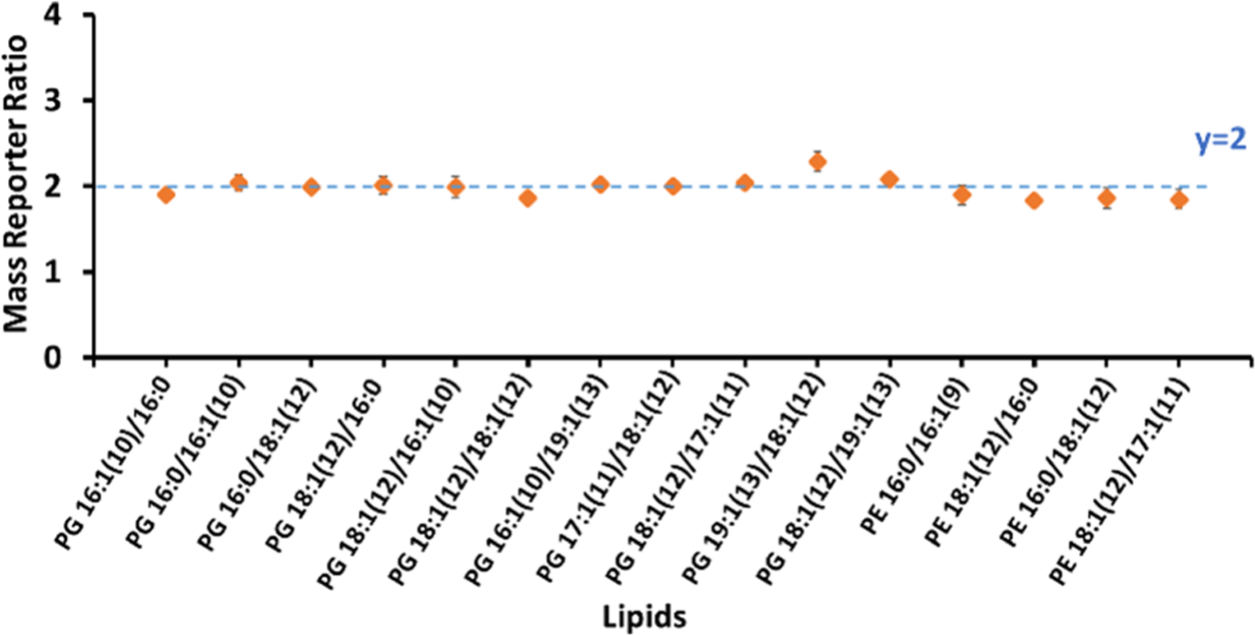
Measured mass reporter intensity ratios of the 15 lipids with assigned *sn*-positions, represented by orange squares, accurately reflect their relative concentrations in the two *E. coli* samples, which were mixed at a concentration ratio of 2. The dashed blue line signifies a theoretical mass reporter ratio of 2 for each lipid.

Two isobaric tags, iTRAQ-114 and 117, were used to individually label the two aforementioned aziridine lipid samples extracted from *E. coli*. Subsequent to labeling, the samples were combined and subjected to nano-ESI-MS/MS analysis in positive ion mode (Figure 4c). Due to the high labeling efficiency through the NHS chemistry, both amino groups in the aziridine ring and headgroup of PE lipids were labeled after 6 h at 50 °C. As expected, lipids such as PE 34:2, PE 36:2, PG 34:2, and PG 36:2 produced not only monotagged aziridinated products but also ditagged aziridinated PG lipids (diazi) and tritagged aziridinated PE lipids. After lithiation, *sn*-positional diagnostic ions were detected for isomer identification upon CID of the lithiated tagged lipid (Figure S12). To obtain relative quantities of lipid *sn*-positional isomers, HCD in MS^4^ was performed in an HPLC-MS analysis. Due to the matrix effect inherent in complex biological samples, the detection of mass reporter ions after MS^4^ with a shotgun approach (nano-ESI-MS/MS) proved challenging. To aid in the formation of lithium adducts, we introduced lithium acetate (4 mM) into the flow path postcolumn via a tee-union at a flow rate of 20 μL/min. The ratios of reporter ions at *m*/*z* = 114 and 117 were obtained for lipid quantification at the *sn*-positional isomer level (Figure 5). These ratios indicate the relative concentration ratios of 2 between the two solutions of lipid *sn*-positional isomers, achieving an accuracy ranging from 87 to 97.5%. In total, 15 lipid *sn*-positional isomers were precisely quantified, underscoring the method’s ability to differentiate and measure lipid isomers, even within complex biological samples.

### Enhanced Relative Quantification of Lipid Isomers in Colon Cancer and Healthy Human Plasma

Colon cancer is the second most common cause of cancer-related deaths.^45^ Understanding the alterations in lipid concentrations during the initiation and progression of colon cancer is crucial for identifying potential biomarkers for disease diagnosis and monitoring. Previous literature has reported PC lipid profile changes in the colon cancer.^46^ However, it is still challenging to characterize the PC lipid profile at the *sn*-positional isomer level which is involved in cell remodeling to adapt to the cancer environment. In this work, we apply our method to identify PC lipids from colon cancer human plasma at the *sn*-positional isomer level and observe the abundance changes compared to those of lipids from healthy human plasma.

**Figure 6:**
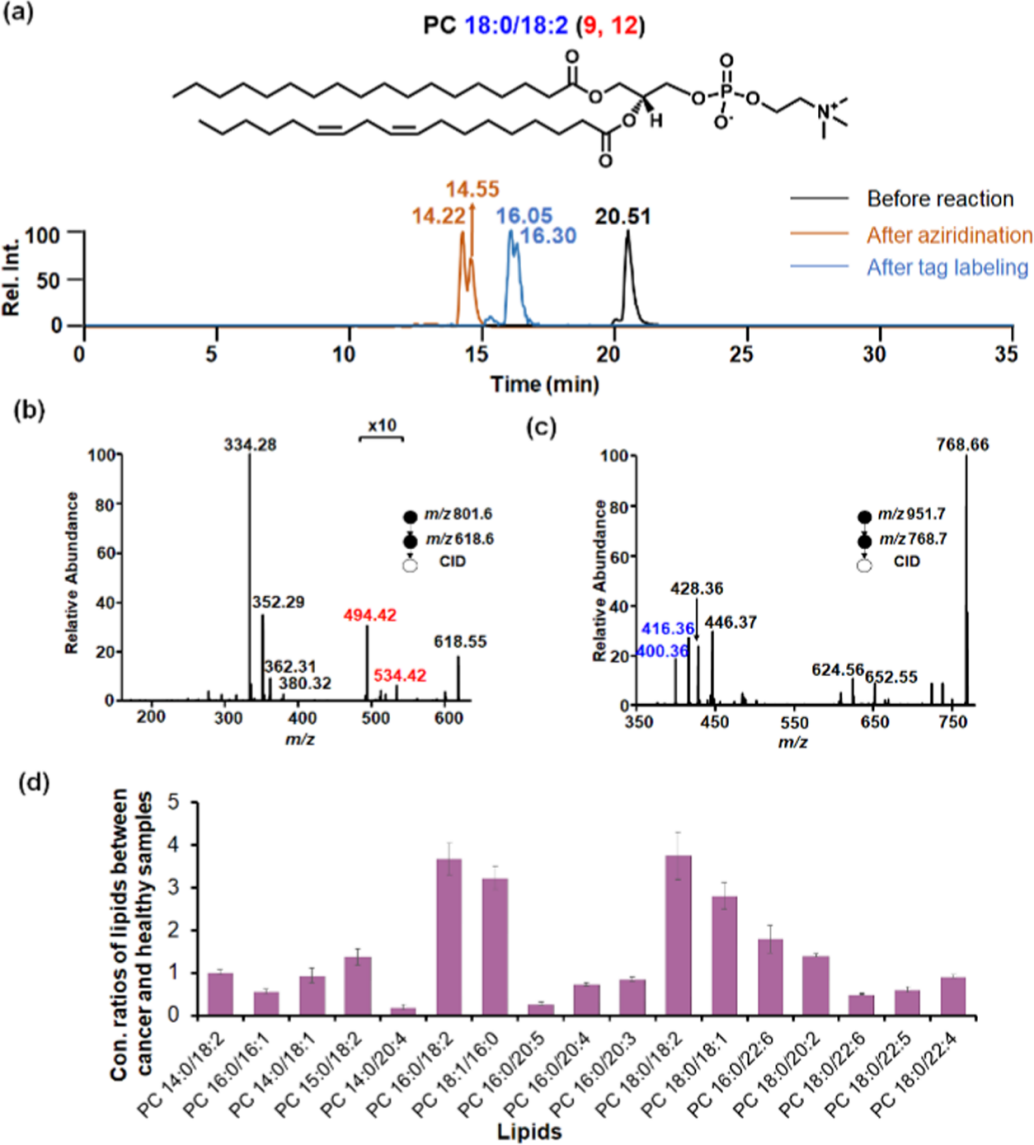
(a) Extracted ion chromatograms of PC 36:2 before reaction, after aziridination, and after isobaric tag labeling; (b) Tandem mass spectrum of PC 36:2 lipid aziridine. (c) Tandem mass spectrum of isobaric tag-labeled PC 36:2, showing the acyl chain 18:2 at the *sn*-2 position; (d)Concentration ratios of PC lipids from colon cancer and healthy human plasma samples, determined after identifying *sn*-positional isomers. Triplicates were performed to generate the error bars. C=C bond location diagnostic ions are labeled in red, and *sn*-positional diagnostic ions are labeled in blue.

PC lipids were extracted from colon cancer cells and healthy human plasma separately. Aziridination and tag labeling were performed under the same reaction conditions as described before. The lipid extracts from colon cancer and healthy human plasma were labeled with isobaric tags-114 and -117, respectively. The mixture of the two tag-labeled extracts was then analyzed by HPLC with postcolumn lithiation coupled with MS/MS. The determination of PC lipids with the resolution of both C=C bond positions and *sn*-positions was achieved by CID of lipid aziridines and lithiated tagged lipids. Taking PC 36:2 as an example, the isomer mixture was detected at a retention time of 20.51 min before reaction (Figure 6a). After aziridination, the monoaziridine products at *m*/*z* 801.6 were observed at the retention times of 14.22 and 14.55 min, showing that aziridination occurred at any of the two C=C bonds in this lipid. Upon CID, fragments at *m*/*z* 334 and 352 indicated the presence of acyl chains 18:0 and 18:2 in the lipid (Figure 6b). Additionally, diagnostic ions for C=C locations were observed at *m*/*z* 494.4 and 534.4, indicating the C=C bonds at Δ9 and Δ12 in the acyl chain of 18:2, i.e., PC 18:0_18:2(9, 12). After isobaric tag labeling and lithiation, the lithiated tagged lipid produced *sn*-positional diagnostic ions at *m*/*z* 400.4 and 416.4 in MS^3^, showing the acyl chain 18:0 at the *sn*-1 position and 18:2 at the *sn*-2 position, i.e., PC 18:0/18:2 (Figure 6c).

Quantification of this lipid at the *sn*-positional isomer level was achieved by releasing the mass reporter ion upon HCD of the *sn*-diagnostic ion at *m*/*z* 400.36. The relative concentration of each lipid from colon cancer plasma and healthy human plasma was achieved by calculating the intensity ratios of mass reporters at *m*/*z* = 114 and 117. The concentration of PC 18:0/18:2 in colon cancer plasma was 3.74 times higher than that in healthy human plasma (Figure 6d). In total, we identified and quantified 17 lipid *sn*-positional isomers simultaneously (Table S1). Among these, 7 PC lipid isomers, namely, PC 14:0/20:4, PC 16:0/18:2, PC 18:1/16:0, PC 16:0/20:5, PC 18:0/18:2, PC 18:0/18:1, and PC 18:0/22:6, exhibited significant abundance changes in colon cancer plasma, either exceeding or falling below 2-fold compared to their counterparts in healthy human plasma (Figure 6d).

## Conclusions

In summary, we have developed an aziridine-based isobaric tag labeling strategy that achieves relative quantification of unsaturated *sn*-positional GPLs with simultaneous resolution of lipid *sn*-positions and C=C bond positions. After aziridination, isobaric tag labeling, and lithiation steps, lithiated tagged lipids can be formed. Upon CID, *sn*-positional diagnostic ions are detected for *sn*-positional isomer identification. Notably, these *sn*-positional diagnostic fragments carry isobaric tags, which can further release mass reporter ions upon HCD for isomer quantitation purposes. C=C bond positions in lipids can be elucidated by CID of lipid aziridine to generate C=C bond diagnostic ions. The methodology’s capability to accurately quantify *sn*-positional isomers has been demonstrated using lipid standards, including PC, PE, and PG. Furthermore, the analysis of *E. coli* lipid extracts showed that the measured intensity ratios of mass reporters released from *sn*-positional diagnostic ions accurately revealed concentration ratios of lipid isomers using this method. Besides, the strategy was also applied to human colon cancer plasma, and relative quantification of lipid *sn*-positional isomers was obtained with 7 PC lipids that showed significant concentration changes in the colon cancer plasma. The method exhibits great potential for advancing research in the field of disease-related lipid isomers.

## Supporting Information Available

Additional full mass spectra and tandem mass spectra, diagnostic ions, and detection limit experimental details (PDF)
