# Supplementary material for: Lipid Isobaric Mass Tagging for Enhanced Relative Quantification of Unsaturated sn-Positional Isomers
Source: ACS Meas Sci Au. 2024 Jan 11;4(2):213–22. doi: 10.1021/acsmeasuresciau.3c00062 (PMC11027206; doi:10.1021/acsmeasuresciau.3c00062)
Supplement: Supplementary file 1 — tg3c00062_si_001.pdf [file tg3c00062_si_001.pdf]

Supporting Information

Lipid Isobaric Mass Tagging for Enhanced Relative Quantification of Unsaturated *sn*-Positional Isomers

Tingyuan Yang,[a] Shuli Tang,[a] Jiaxin Feng,[a] Xin Yan*[a]

[a] Department of Chemistry, Texas A&M University, 580 Ross St., College Station, TX 77843, USA

*Corresponding author: xyan@tamu.edu

Table of Content

S1. Structures of *sn*-positional diagnostic ions of PC 16:0/18:1 and PC 18:1/16:0

S2. Tandem mass spectra of sodiated tagged PC 16:0/18:1 and PC 18:1/16:0

S3. Reaction optimization of aziridination of PC 16:0/20:4 using different concentrations of pyridine, HOSA, and catalyst, and applying different reaction times

S4. Full mass spectrum of iTRAQ-labeling reaction of PC 18:1/16:0

S5. Limit of detection

S6. MS^3 of lithiated isobaric tag labeled PG 18:1/16:0 and PE 18:1/16:0

S7. MS^4 of lithiated isobaric tag labeled PG 18:1/16:0 and PE 18:1/16:0

S8. Calibration curves of PG, PE and PC for relative quantification at the lipid level and *sn*-positional isomer level

S9. Full mass spectrum of iTRAQ-labeling reaction of PE 18:1/16:0

S10. Full mass spectrum of iTRAQ-labeling reaction of PC 16:0/18:2

S11. Tandem mass spectra of lipid aziridines for identifying lipid C=C positional isomers in E. coli lipid extract

S12. Tandem mass spectra of lipid aziridines for identifying lipid *sn*-positional isomers in E. coli lipid extract

S13. *sn*-positional diagnostic ions and relative quantification of lipid isomers in colon cancer human plasma and healthy human plasma

S1. Structures of *sn*-positional diagnostic ions of PC 16:0/18:1 and PC 18:1/16:0

sn-positional diagnostic ions for PC 16:0/18:1

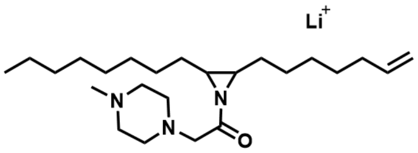

***m/z*: 398.3717**

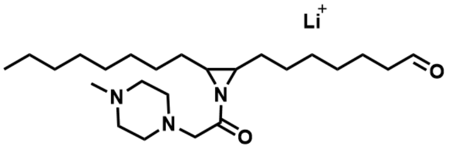

***m/z*: 414.3666**

sn-positional diagnostic ion for PC 18:1/16:0

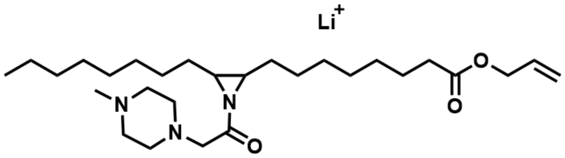

***m/z*: 484.4085**

Figure S1. Structures of *sn*-positional diagnostic ions of PC 16:0/18:1 and PC 18:1/16:0

S2. Tandem mass spectra of sodiated tagged PC 16:0/18:1 and PC 18:1/16:0

Sodium ion adduction was also studied after isobaric tag labeling. In this experiment, diagnostic ions for C=C bond locations and *sn*-positions were detected during MS^3 (Figure S2). However, the MS^4 spectrum did not reveal any mass reporter ions when fragmenting the diagnostic ions for *sn*-positions. Conversely, lithium ion-adducted lipids allowed for the observation of reporter ions in MS^4, attributed to the elevated intensities of lithiated diagnostic ions for *sn*-positions in MS^3. Equal concentrations of lithium salt and sodium salt were employed. If sodium is to be used in the method, a higher concentration of sodium salt may be required.

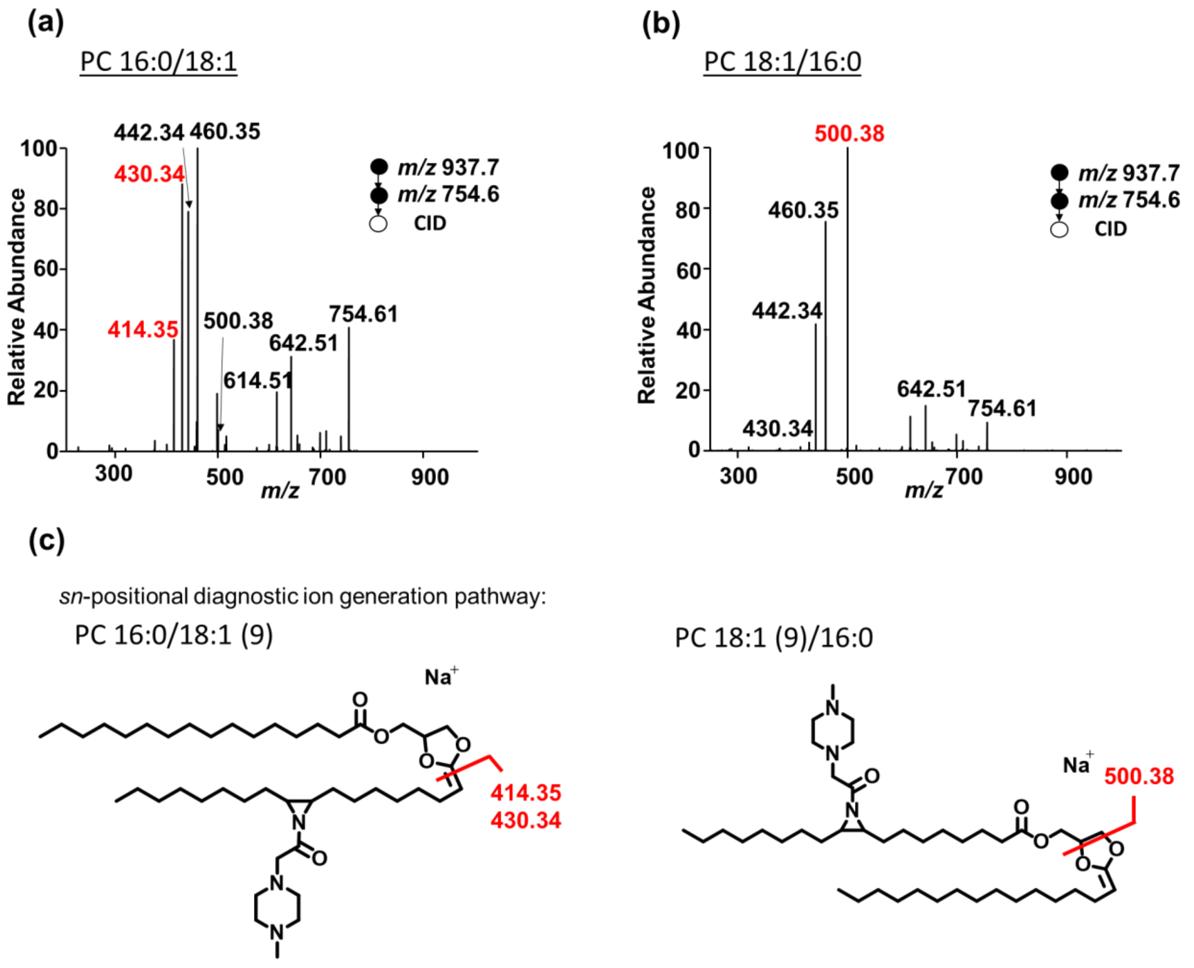

Figure S2. Tandem mass spectra of sodiated tagged (a) PC 16:0/18:1 and (b) PC 18:1/16:0. (c) Generation of *sn*-positional diagnostic ion upon CID. Diagnostic ions are labeled in red.

S3. Reaction optimization of aziridination of PC 16:0/20:4 using different concentrations of pyridine, HOSA, and catalyst, and applying different reaction times

100 µM lipid PC 16:0/20:4 was used for reaction optimization. The conversion distributions of the mono-aziridination product (mono-azi), di-aziridination product (di-azi), tri-aziridination product (tri-azi), and tetra-aziridination product (tetra-azi) were calculated by taking the intensity of the specific product intensity over the sum intensity of all lipid aziridines. The results indicate that the di-azi product was dominant and achieved the highest conversion under the reaction condition of 5 equivalent (eq) of pyridine, 1.5 eq of HOSA, 20% eq catalyst, and 24 hours of reaction time (Figure S3a-d).

In polyunsaturated lipids, such as PC 16:0/20:4, aziridination (azi) occurred at all C=C bonds, resulting in the formation of mono-azi and di-azi products. Subsequent MS^3 analysis of the mono-azi lipid revealed diagnostic ions indicating C=C bond positions at $\Delta5$, $\Delta8$, $\Delta11$, and $\Delta14$ (Figure S3e). The intensities of these diagnostic ions suggested varying reaction efficiencies at different C=C locations. For instance, the C=C bond at $\Delta5$, situated closest to the carbonyl group, exhibited the lowest abundance of diagnostic ion peaks, possibly due to reduced reaction efficiency at this position caused by steric hindrance from the head group. Importantly, the variability in reaction efficiencies at different C=C bond positions does not impact relative quantification, as our method assesses abundance changes of the same lipid across different samples with identical reaction efficiencies.

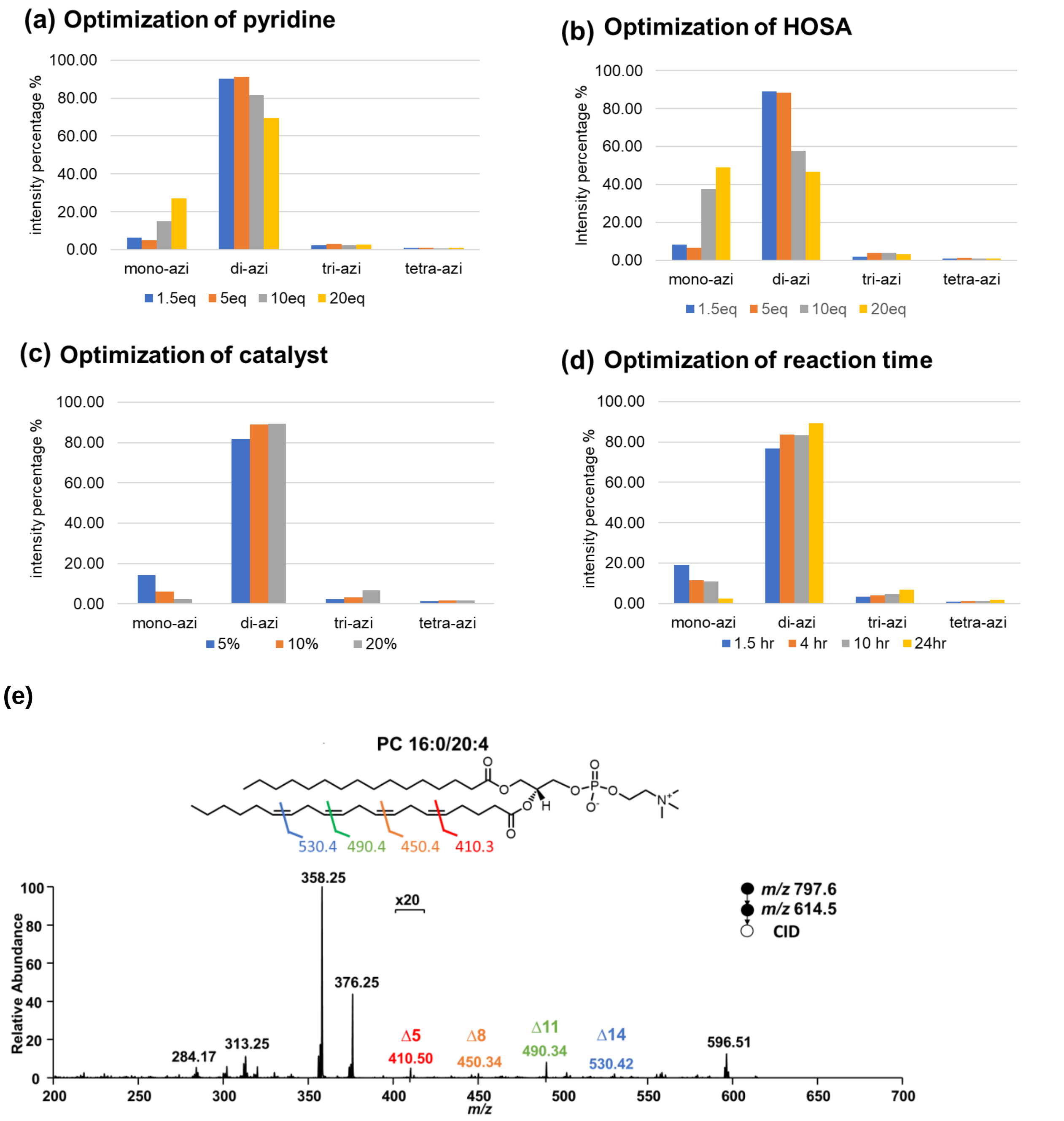

Figure S3. The reaction optimization of aziridination using different eq of (a) pyridine, (b) HOSA, (c) catalyst, and (d) applying different reaction times. (e) Tandem spectrum of PC 16:0/20:4 mono-aziridine product.

In many reactions, there are notable variations in the efficiency of *cis* and *trans* C=C bonds. However, we specifically chose *N-H* aziridination to label lipid C=C bonds, as this

method exhibits minimal disparity in reaction efficiencies, as demonstrated in the referenced paper (40. *Angew. Chem. Int. Ed.* **2017**, *56*, 9886–9890). For instance, in Compound 7 with a *trans* C=C bond, the aziridination yield is 80%, while in Compound 28 with a *cis* C=C bond, the yield is 77%. These comparable yields underscore the uniform reaction efficiency of both *cis* and *trans* compounds, alleviating concerns in our analysis using the chosen tagging method.

N-H aziridination of lipid C=C bonds has been successfully demonstrated across various lipid classes, including triglycerides, fatty acids, fatty acid esters, phospholipids, and cholesterol ester lipids as presented in this study and in our previous work (*Angew. Chem. Int. Ed.* **2022,** *61*, e202207098). This collective evidence underscores the versatility of aziridination as a derivatization method for unsaturated lipids.

Differences in aziridination efficiencies were observed across various lipid classes. For instance, the aziridination yields for PE 32:1 and PG 32:1 were 77% and 94%, respectively, calculated as [Intensity of lipid aziridine]/([Intensity of lipid aziridine] + [Intensity of unreacted lipid]). It is important to emphasize that while there is variability in reaction efficiencies among different lipid classes, this does not affect relative quantification. Our method evaluates changes in abundance for the same lipid across different samples with consistent reaction efficiencies.

S4. Full mass spectrum of iTRAQ-labeling reaction of PC 18:1/16:0

PC 18:1/16:0 aziridine reacted with 50 eq iTRAQ at 50 degrees Celsius for 6 hours. After the reaction, protonated tag-labeled lipid was detected at *m/z* 919.7 while lipid aziridine (*m/z* 775.6) was not observed.

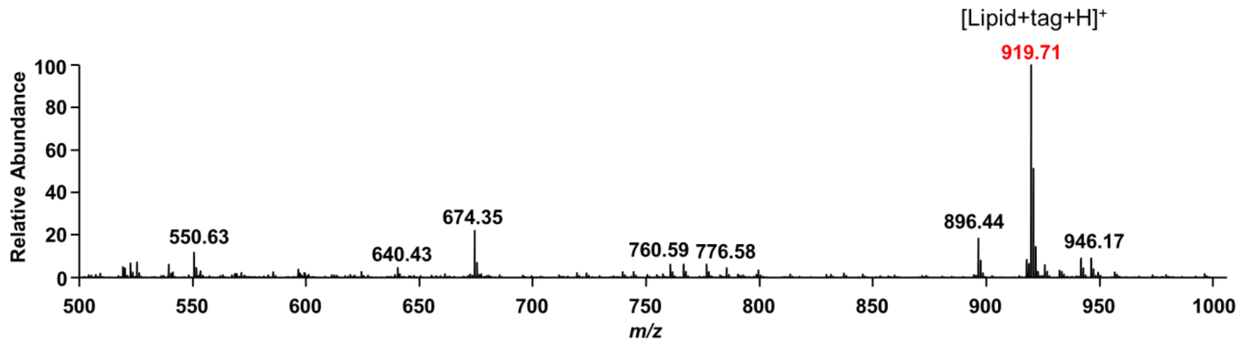

Figure S4. Full mass spectrum of iTRAQ-labeling reaction mixture of PC 18:1/16:0 under the optimized condition. The product peak was labeled in red.

S5. Limit of detection

The detection limit of the method was evaluated using PC 18:1/16:0, PG 16:0/18:1 and PE 16:0/18:1. Following aziridination, isobaric tag labeling, and lithiation, samples were introduced into MS for analysis at concentrations of 1 nM, 10 nM, 100 nM, 500 nM, 1 µM, 5 µM, and 10 µM. The detection limit for PC 18:1/16:0 was determined at 1 µM, evidenced by the presence of the mass reporter ion at *m/z* 114.1 in the MS^4 spectrum, with a signal-to-noise ratio of 87. However, the reporter ion was not discernible with a signal-to-noise ratio higher than 3 at 500 nM. Similar assessments for PG 16:0/18:1 and PE 16:0/18:1 indicated a detection limit of 5 µM for both.

(a) PC 18:1/16:0

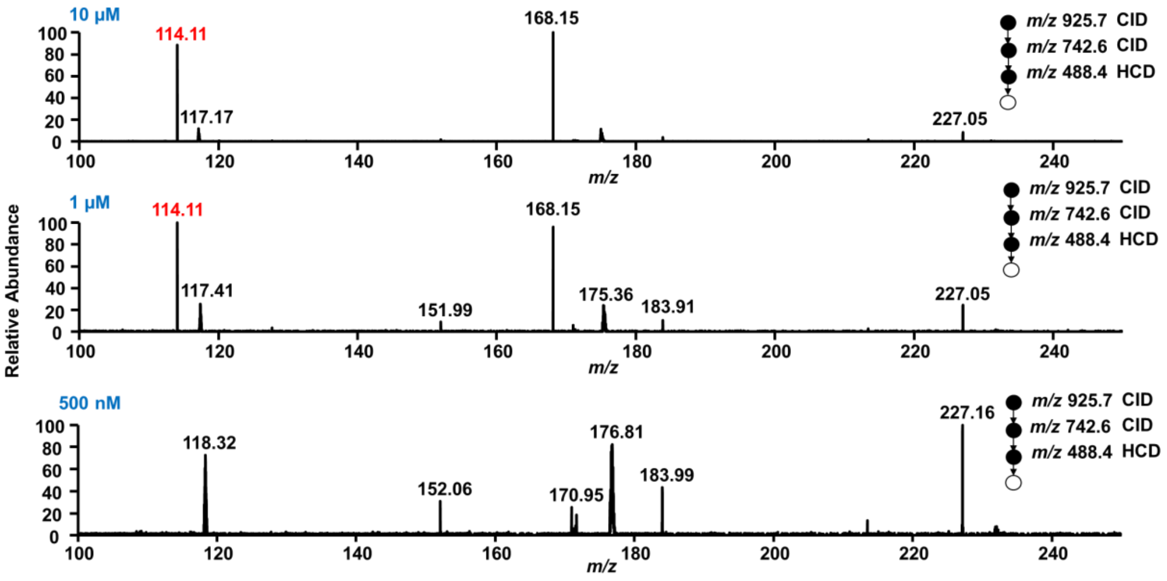

(b) PE 16:0/18:1

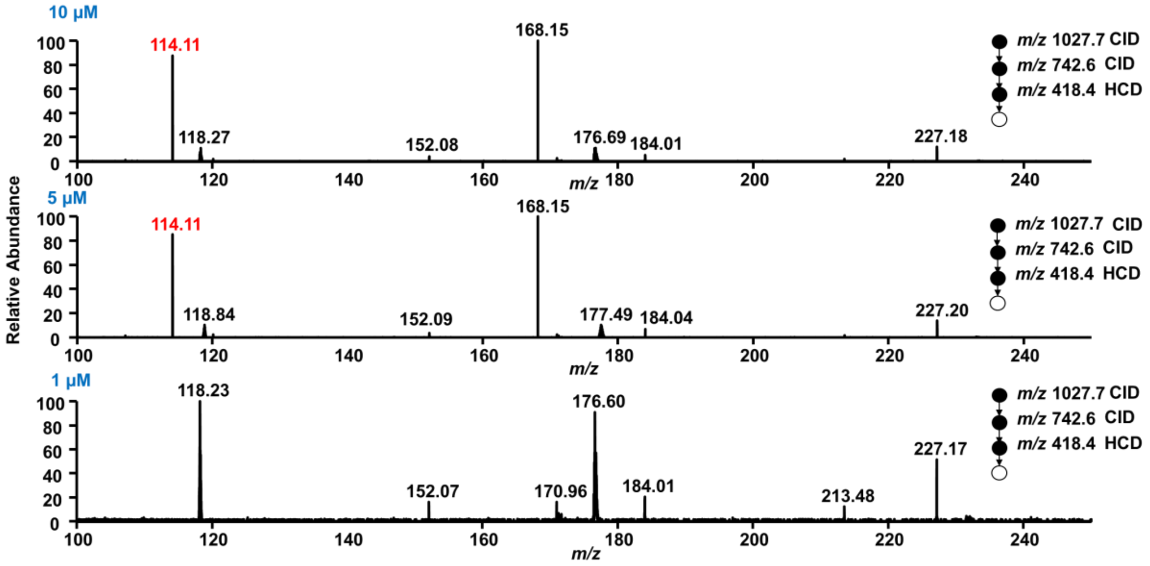

(c) PG 16:0/18:1

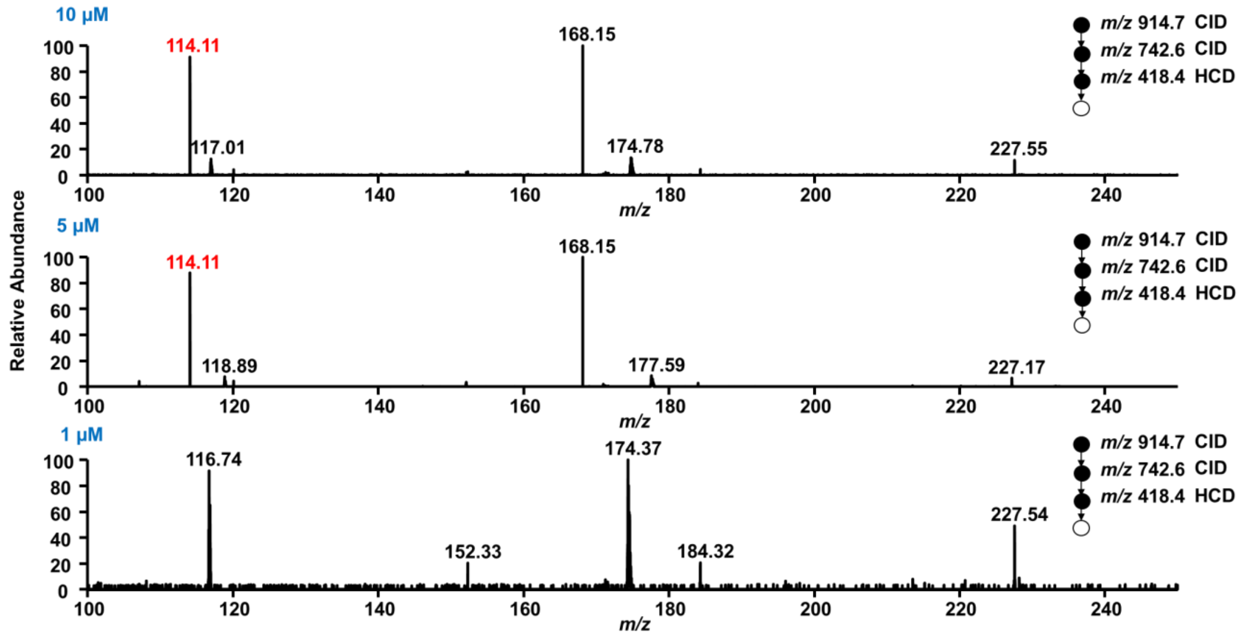

Figure S5. Tandem mass spectra of lithiated tag-labeled (a) PC 18:1/16:0 at concentrations of 10 μM, 1 μM, and 500 nM, (b) PE 16:0/18:1 at concentrations of 10 μM, 5 μM, and 1 μM, and (c) PG 16:0/18:1 at concentrations of 10 μM, 5 μM, and 1 μM. Mass reporter ion at *m/z* 114.11 was detected and labeled in red.

S6. MS^3 of lithiated isobaric tag labeled PG 18:1/16:0 and PE 18:1/16:0

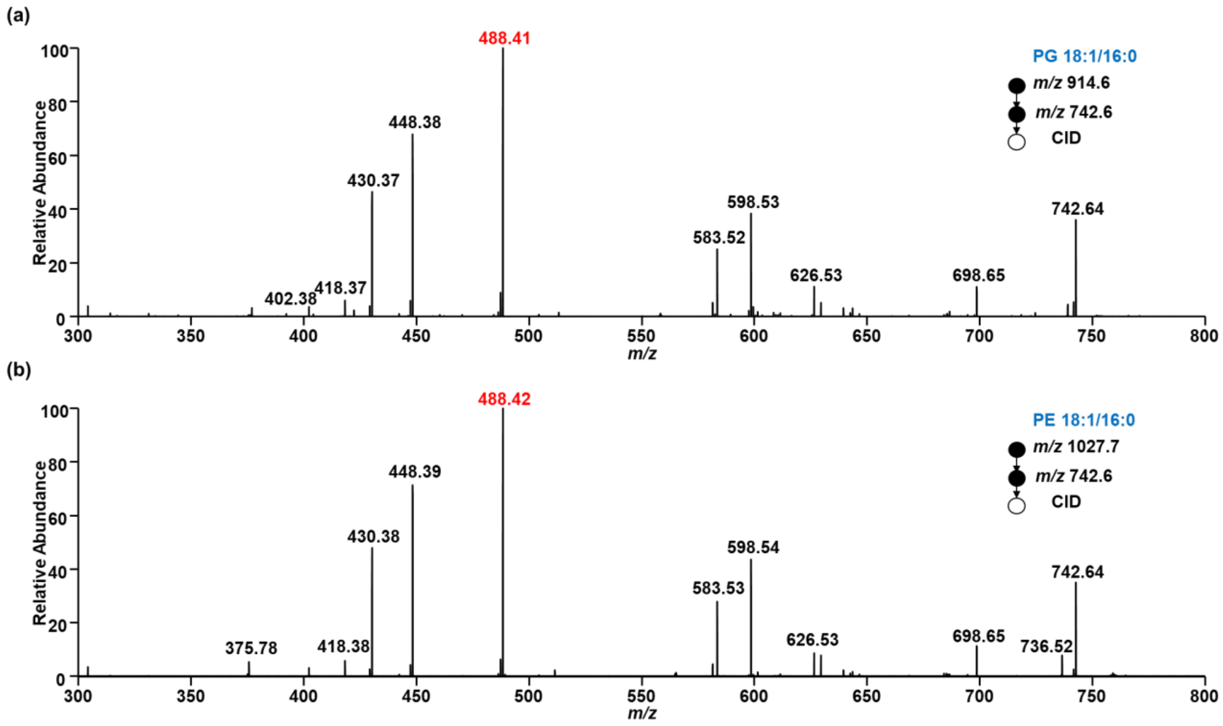

Figure S6. MS^3 spectra of lithiated isobaric tag labeled lipids (a) PG 18:1/16:0, (b) PE 18:1/16:0. The *sn*-positional diagnostic ion was observed at *m/z* 488.4 and labeled in red.

S7. MS^4 of lithiated isobaric tag labeled PG 18:1/16:0 and PE 18:1/16:0

Two samples containing both PG 18:1/16:0 and PE 18:1/16:0 were prepared with a total concentration ratio of 1:2. After aziridination, one sample was labeled with iTRAQ-114 while the other was labeled with iTRAQ-117. After mixing the two samples and adding lithium acetate, the mixture was injected into MS for analysis.

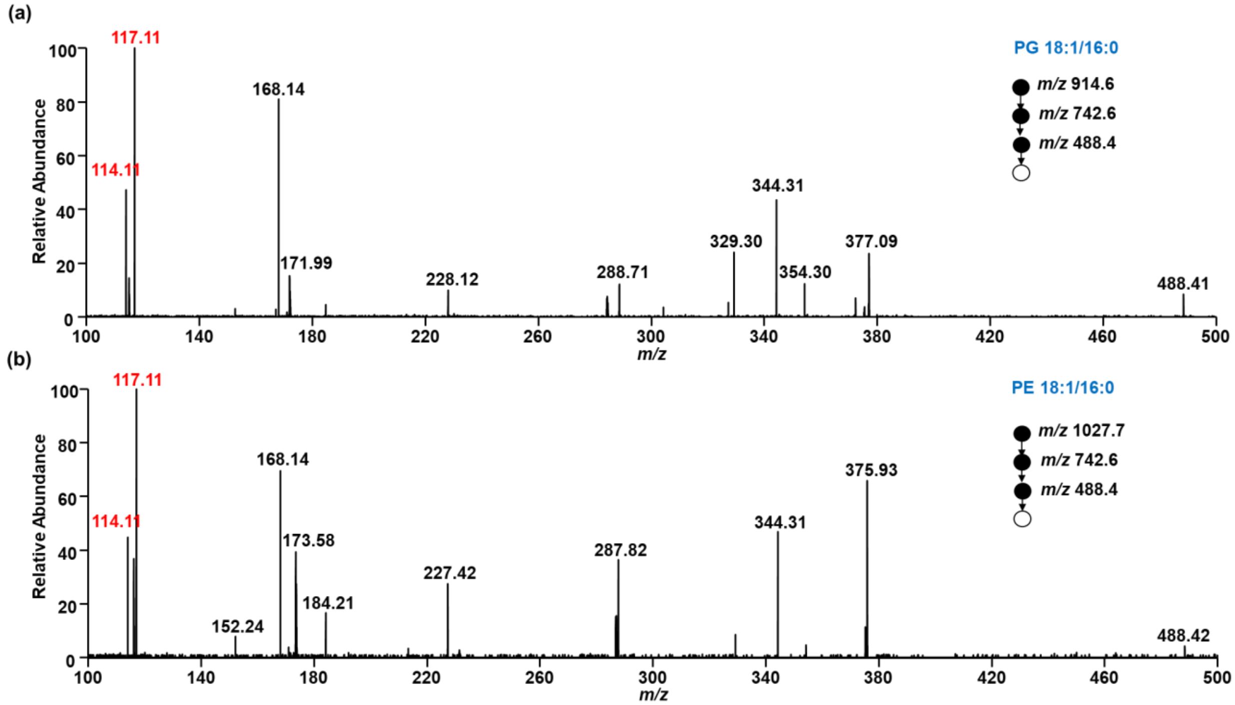

Figure S7. (a) MS^4 spectra of lithiated isobaric tagged (a) PG 18:1/16:0 and (b) PE 18:1/16:0 after mixing the two samples. The mass reporter ions at *m/z* 114 and 117 were observed and the mass reporter ion intensity ratio (I114/I117) indicated the concentration ratio of the two lipids at 1:2. Mass reporter ions are labeled in red.

S8. Calibration curves of PG, PE and PC for relative quantification at the lipid level and *sn*-positional isomer level

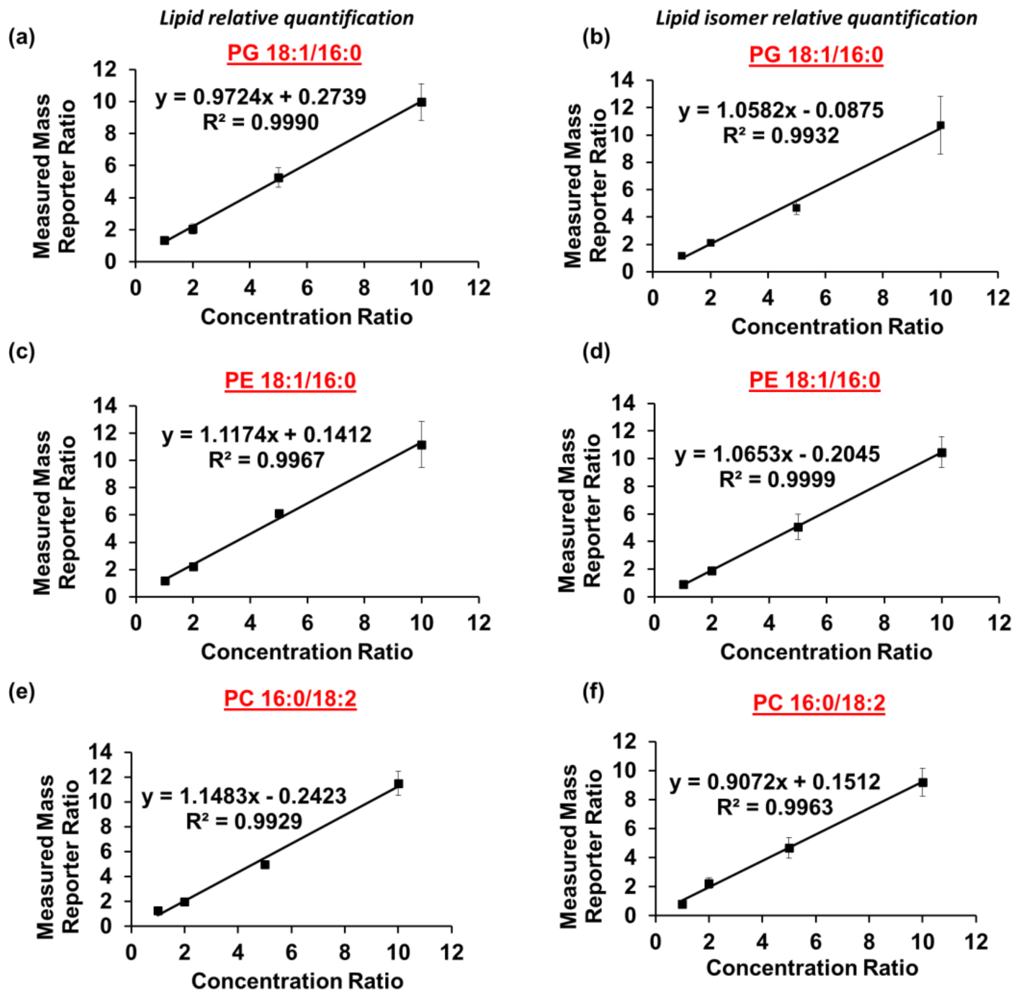

Figure S8. Linear correlation between the measured intensity ratios of reporter ions (I114: I117) and the concentration ratios of tag-labeled lipids from lipid sample solutions and control solutions for quantification of (a) PG 18:1/16:0 at the lipid level, (b) PG 18:1/16:0 at the isomer level, (c) PE 18:1/16:0 at the lipid level, (d) PE 18:1/16:0 at the isomer level, (e) PC 16:0/18:2 at the lipid level, and (f) PC 16:0/18:2 at the isomer level. Triplicates were performed to generate the error bars.

S9. Full mass spectrum of iTRAQ-labeling reaction of PE 18:1/16:0

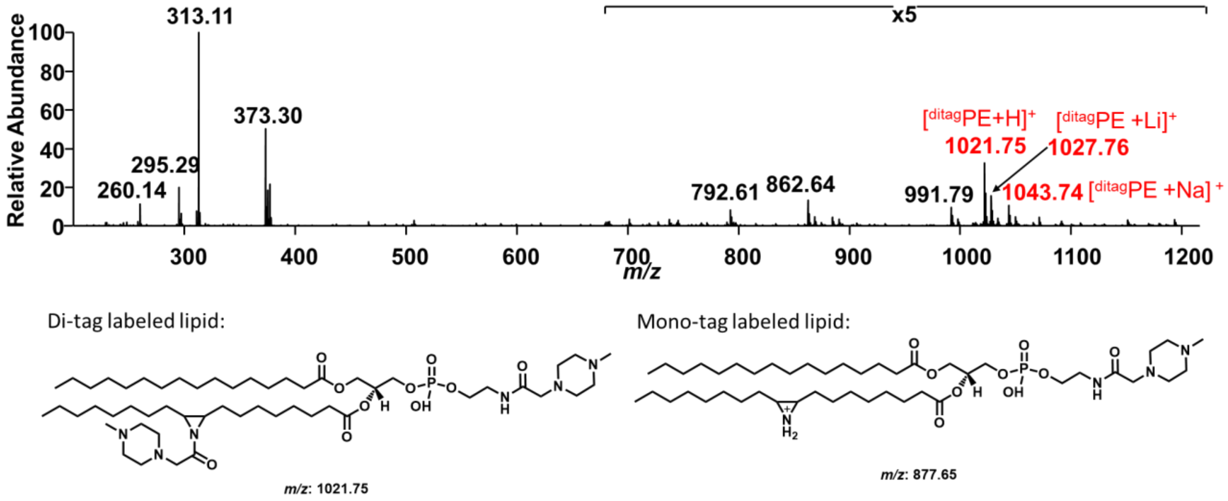

Figure S9. Full mass spectrum of iTRAQ-labeling reaction of PE 18:1/16:0.

S10. Full mass spectrum of iTRAQ-labeling reaction of PC 16:0/18:2

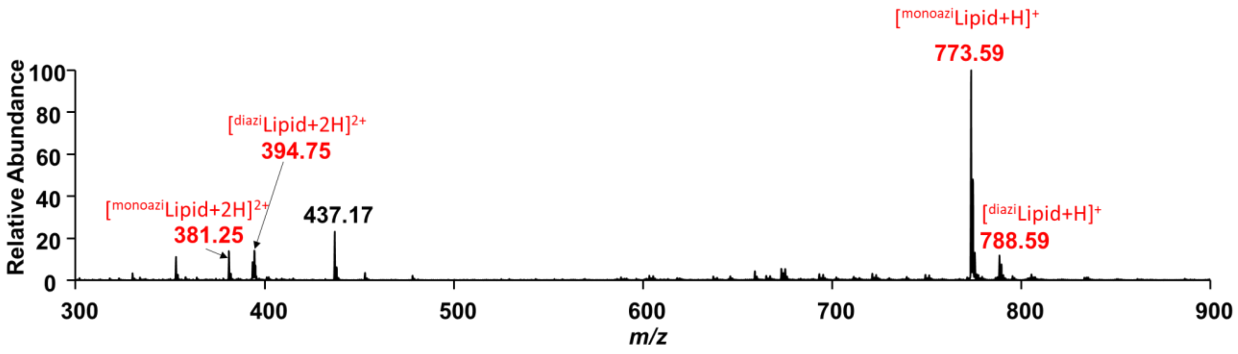

Figure S10. Full mass spectrum of iTRAQ-labeling reaction of PC 16:0/18:2

S11. Tandem mass spectra of lipid aziridines for identifying lipid C=C positional isomers in E. coli lipid extract

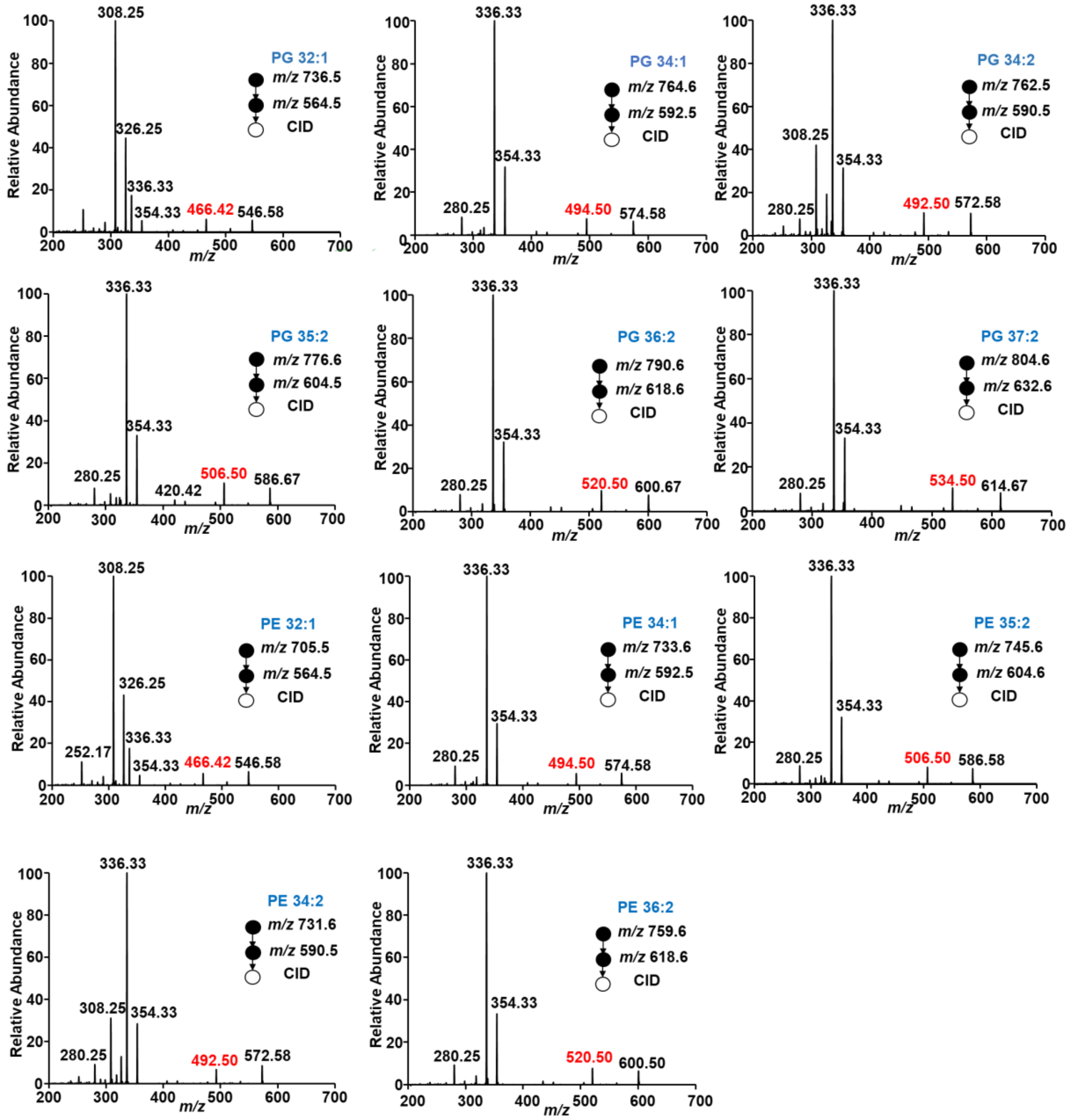

Figure S11. Tandem mass spectra of lipid aziridines for identifying lipid C=C positional isomers. C=C bond diagnostic ions were labeled in red.

S12. Tandem mass spectra of lipid aziridines for identifying lipid *sn*-positional isomers in E. coli lipid extract.

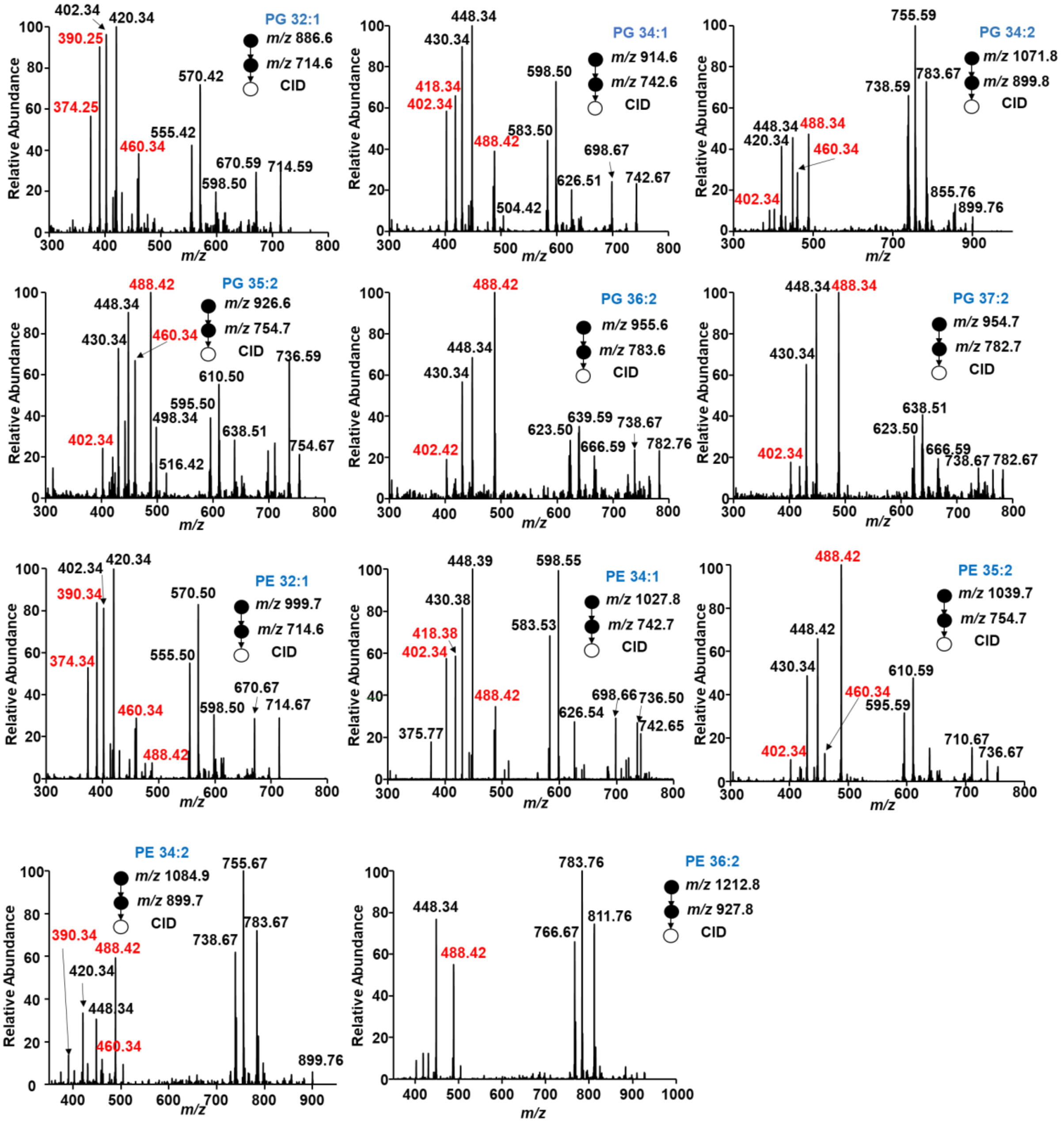

Figure S12. Tandem mass spectra of lipid aziridines for identifying lipid *sn*-positional isomers. *Sn*-positional diagnostic ions were labeled in red.

S13. *sn*-Positional diagnostic ions and relative quantification of lipid isomers in colon cancer human plasma vs. healthy human plasma.

In MS analysis, different stages, namely MS^2, MS3, and MS^4, were used for different levels of lipid structure and quantification analysis. MS^2 enabled the detection of phospholipids' head group loss peaks, allowing identification of a range of phospholipids in the sample. Following aziridination and tag labeling, *sn*-positional diagnostic ions were observed with lithium-ion adduction during MS^3. Subsequently, MS^4 was executed, releasing *sn*-positional diagnostic ions and mass reporter ions for relative quantification. When coupling with HPLC, the DDA acquisition was employed for MS^2 data collection. Given the need for additional tandem MS, manual settings for MS^3 and MS^4 were incorporated into the MS acquisition methods, targeting specific lipids and their corresponding retention times. In short, our approach involves untargeted MS^2 data acquisition, while MS^3 and MS^4 analyses are guided by lipid information provided in the MS^2 stage.

Table S1. *sn*-positional diagnostic ions and relative quantification of lipid isomers in colon cancer human plasma and healthy human plasma.

Lipids	Identified PCs	*sn*-position diagnostic ions	Relative quantification
PC 32:2	PC 14:0/18:2(9, 12)	400.3627	1.01
PC 32:1	PC 16:0/16:1(7)<br>PC 16:0/16:1(9)	374.3483	0.56
	PC 14:0/18:1(9)<br>PC 14:0/18:1(11)	402.3802	0.94
PC 33:2	PC 15:0/18:2(9, 12)	400.3627	1.37
PC 34:4	PC 14:0/20:4(5, 8, 11, 14)	424.3644	0.20
PC 34:2	PC 16:0/18:2(9, 12)<br>PC 16:0/18:2(9, 14)	400.3643	3.66
PC 34:1	PC 18:1(9)/16:0<br>PC 18:1(11)/16:0<br>PC 18:1(14)/16:0	488.4168	3.22
PC 36:5	PC 16:0/20:5(5, 8, 11, 14, 17)	422.3484	0.27
PC 36:4	PC 16:0/20:4(5, 8, 11, 14)	424.3644	0.73
PC 36:3	PC 16:0/20:3(9, 11, 14)	426.3801	0.85
PC 36:2	PC 18:0/18:2(9, 12)	400.3642	3.74
PC 36:1	PC 18:0/18:1(9)	402.3793	2.80
PC 38:6	PC 16:0/22:6(4, 7, 10, 13, 16, 19)	448.3641	1.79
PC 38:2	PC 18:0/20:2(11, 14)	428.3951	1.40
PC 40:6	PC 18:0/22:6(4, 7, 10, 13, 16, 19)	448.3641	0.50
PC 40:5	PC 18:0/22:5(7, 10, 13, 16, 19)	450.3797	0.61
PC 40:4	PC 18:0/22:4(7, 10, 13, 16)	452.3593	0.91

Note: Due to the relatively high limit of detection of the method, lipids with low abundance may not achieve isomer quantification. For example, PC 36:2, besides PC 18:0/18:2 that we have already identified (see Figure 7), we also detected PC 16:0_20:2. Upon CID of lipid aziridine, peaks at *m/z* 362.31 and 380.32 were observed, revealing that the fatty acyl chains were C16:0 and 20:2 (Figure 7b). However, due to its low abundance in the sample, *sn*-positional diagnostic ion peaks at *m/z* 428.39 and 444.39 could not be observed (Figure 7c).
